# Delimiting species of *Protaphorura* (Collembola: Onychiuridae): integrative evidence based on morphology, DNA sequences and geography

**DOI:** 10.1038/s41598-017-08381-4

**Published:** 2017-08-15

**Authors:** Xin Sun, Feng Zhang, Yinhuan Ding, Thomas W. Davies, Yu Li, Donghui Wu

**Affiliations:** 1 Key Laboratory of Wetland Ecology and Environment, Northeast Institute of Geography and Agroecology, Chinese Academy of Sciences, Changchun, 130102 China; 2 Department of Entomology, College of Plant Protection, Nanjing Agricultural University, Nanjing, 210095 China; 3 Engineering Research Center of Chinese Ministry of Education for Edible and Medicinal Fungi, Jilin Agricultural University, Changchun, 130118 China; 4 Key Laboratory of Zoological Systematics and Evolution, Institute of Zoology, Chinese Academy of Sciences, Beijing, 100101 China; 5 Centre for Geography, Environment and Society, University of Exeter, Penryn, Cornwall, TR10 9FE UK; 6 Key laboratory for vegetation ecology, ministry of education, Northeast Normal University, Changchun, 130117 China; 7 Jilin Provincial Key Laboratory of Animal Resource Conservation and Utilization, Northeast Normal University, Changchun, 130117 China

## Abstract

Species delimitation remains a significant challenge when the diagnostic morphological characters are limited. Integrative taxonomy was applied to the genus *Protaphorura* (Collembola: Onychiuridae), which is one of most difficult soil animals to distinguish taxonomically. Three delimitation approaches (morphology, molecular markers and geography) were applied providing rigorous species validation criteria with an acceptably low error rate. Multiple molecular approaches, including distance- and evolutionary model-based methods, were used to determine species boundaries based on 144 standard barcode sequences. Twenty-two molecular putative species were consistently recovered across molecular and geographical analyses. Geographic criteria were was proved to be an efficient delimitation method for onychiurids. Further morphological examination, based on the combination of the number of pseudocelli, parapseudocelli and ventral mesothoracic chaetae, confirmed 18 taxa of 22 molecular units, with six of them described as new species. These characters were found to be of high taxonomical value. This study highlights the potential benefits of integrative taxonomy, particularly simultaneous use of molecular/geographical tools, as a powerful way of ascertaining the true diversity of the Onychiuridae. Our study also highlights that discovering new morphological characters remains central to achieving a full understanding of collembolan taxonomy.

## Introduction

Springtails (Collembola) are one of the dominant soil arthropods in almost all terrestrial ecosystems. Until now, there have been about 8500 species reported worldwide^1^. Having well differentiated ecomorphological life-forms and feeding guilds, springtails are considered to play important functional roles in many processes of soil ecosystems, such as carbon and nitrogen cycling, soil microstructure formation and plant litter decomposition processes^2–5^. At the same time, the structure of collembolan communities can be greatly influenced by soil acidification, nitrogen supply, global climate change and intensive farming^6, 7^. When combined with recent advances in taxonomy, springtails can provide ideal model systems for many scientific fields (e.g. ecotoxicology, biogeography and ecology)^8^.

The genus *Protaphorura* Absolon, 1901 (Poduromorpha: Onychiuridae) is one of the most abundant collembolan groups which includes more than 130 species^1^, and is widely distributed worldwide. Species of the genus are blind and usually white as other genera of the family^2^. They are mostly euedaphic in different ecosystems, and some species are crop pests which can reduce yields and productivity^9–11^.

Because of frequent variations in morphological characters, species discrimination in *Protaphorura* has been debated for nearly sixty years. The formulae of pseudocelli, which are unique epicuticular structures in Onychiuridae and Tullbergiidae, and probably the defensive structures located on head and body, are proved to be intraspecifically stable in many other genera (e.g. *Thalassaphorura*, *Onychiurus*, *Oligaphorura*, *Dimorphaphorura*) and very useful to differentiate species^12–15^. Unfortunately, the extensive variation of the pseudocelli formula in *Protaphorura* has been disputed as to whether it represents interspecific or intraspecific differences. According to Gisin^16, 17^, adults of European species of the *Onychiurus armatus* group, which is the synonym of the present genus *Protaphorura*, were distinguished from each other mainly by the number of pseudocelli particularly those on the dorsal body. Several new species were reported following Gisin’s definition later^18–20^. However, some authors considered many of Gisin’s species as ecological or local modifications because of the inconsistency in the characters employed^21–23^. Based on Icelandic and Swedish material, Bödvarsson^24, 25^ challenged the Gisin’s system of species discrimination and regarded them as of intraspecific significance, because of the intermediates existing between different forms. To address these problems, Pomorski^26, 27^ studied the postembryonic development of some members of the *P. armata* species group and proposed that the relative position rather than number of pseudocelli is of a high taxonomic value. Besides the formulae of pseudocelli, other diagnostic characters (e.g. the number of chaetae on prothorax, the presence/absence of additional microchaeta on abdominal terga I–III and V, the relative position of prespinal microchaetae on abdominal tergum VI) have been proposed to be useful for species identification. The number and arrangement of parapseudocelli (a similar structure as pseudocelli, but without a chitinized border), which were introduced by Pomorski and are now frequently reported, are considered by some authors to be of doubtful taxonomic value, not just in *Protaphorura*, but even in other Onychiuridae groups^28, 29^. Accordingly, the species boundaries in *Protaphorura* are often ambiguous, particularly for conspecific species reported from different populations^2, 30–33^.

Species delimitation has long been confused because of the differences among contemporary species concepts^34^. Many disciplines and corresponding species concepts, such as the widely used biological species concept, the phylogenetic species concept, and the morphological/phenetic species concept^34^, could be applied to species delimitation, but no single discipline is entirely proper^35^. As a fundamental biological unit, a species needs to be defined upon some properties in a given study. The generalized “independent evolving metapopulation lineage” (Generalized Lineage Concept, GLC)^34, 36^ has been broadly adopted to determine the species boundary from empirical data^37^. In recent years, an integrative approach to taxonomy has been promoted, combining different kinds of disciplines such as morphology, phylogeography, population genetics, ecology and behavioral biology^35, 37, 38^. This could increase rigor in species delimitation, and avoid failure inherent to single disciplines^35^. However, like in most other arthropod groups, the identification and delimitation of Collembola species have relied mainly on the discrimination of external morphological characteristics^8^ and thus species are called as morphospecies. As there are some limitations to the use of the traditional, morphology-based method, other disciplines are often applied to solve taxonomical problems. While some data, like ecology, behavioral biology, life history and chemistry, are relatively difficult to obtain, especially in most cases of Collembola due to their small size and low research effort, DNA taxonomy has been proved particularly powerful when used in combination with traditional taxonomic approaches^39–42^ and increasingly useful for the identification of cryptic species^43–48^. Meanwhile, different delimitation approaches based on distance or evolutionary models have been developed along with advances in DNA sequencing and computational technologies^37^, such as Automatic Barcode Gap Discovery (ABGD)^49^, the Poisson Tree Processes model (PTP)^50^, and the general mixed Yule coalescent model (GMYC)^51^ which were applied for the single genetic locus; the Bayesian method BPP^52^ and O’Meara’s heuristic method^53^ for multiple loci. Every method has its own parameterization and a series of simplifying. ABGD automatically clustered sequences into candidate species based on pairwise distances by detecting barcoding gaps using a command-line version^49^, and PTP tested species boundaries on non-ultrametric phylogenies by detecting significant difference in the number of substitutions between species and within species^50^. In order to strengthen the confidence in the results, applying a wide range of analyses methods to delimit species has been proposed, as any one of the assumptions implicit in using any one method could possibly be violated in a particular empirical system^37^.

DNA-based methods have recently resolved a number of problems encountered when delimiting species in a number of genera of Collembola, e.g. *Orchesella* ^54^, *Deutonura* ^55^, *Lepidocyrtus* and *Pseudosinella* ^56^, *Megalothorax* ^57^, *Entomobrya* ^58^. The only molecular contribution available in the genus *Protaphorura* is from the thesis of Burkhardt^59^. Distance trees of several taxa within the genus were analyzed in his work based on mitochondrial cytochrome oxidase subunits I and II (COI and COII) and the nuclear internal transcribed spacer 1 (ITS1) fragments with concordant patterns obtained from different genes. The ITS1 was less effective for species discrimination than the mitochondrial fragments. The inferences drawn from species delimitation studies should be conservative, and it will be much more convincing if the results are congruent across a wide range of species delimitation approaches applied to the data including molecular, morphological and geographical^37^.

The present study aims to: (i) assess the efficiency of different data (morphology, DNA sequences, geography) and approaches to delimitating species of the genus *Protaphorura*; (ii) evaluate diagnostic utility of morphological characters currently used to distinguish species, and to identify new characters; (iii) summarize a reliable operational workflow for integrative taxonomy of the genus *Protaphorura*, as well as the Onychiuridae in general.

## Results

### Species delimitation

In total, 108 individuals (75% of all barcoded specimens) were examined for morphological diagnostic characters after DNA extraction (Supplementary Table S3). The remaining 36 specimens broke during the extraction or characters were not visible in slide mounted specimens. Ten morphospecies were recognized based on traditional morphological diagnostic characters, including five individuals with either reduced or supplementary pseudocelli developed in one side of the body (Supplementary Tables S3 and S6). Results of examination of other minor additional characters were listed in Supplementary Table S6.

**Figure 1:**
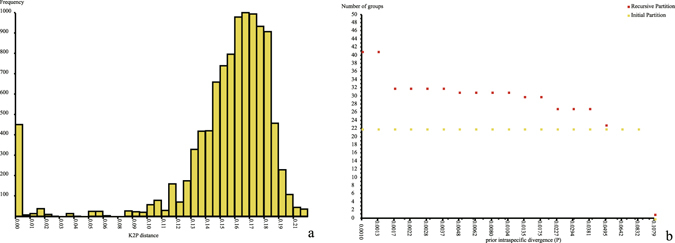
ABGD species delimitation. (**a**) Frequency histogram of K2P pairwise distances. (**b**) Partitions under different prior intraspecific divergences.

ABGD generated 22 initial/primary partitions and 22–41 recursive/secondary partitions while prior intraspecific divergences (P) varied from 0.001 to 0.1079 (Fig. 1b). Rough “barcoding gaps” were observed at K2P distance of around 0.03, 0.045, 0.08 (Fig. 1a), corresponding to plateaus of 27, 23, 22 MOTUs (molecular operational taxonomical units) (Fig. 1b). Primary and secondary partitions were identical at 22 MOTUs (P > 0.064). Because primary partitions are stable on a wider range of prior values and are usually close to the hypothetical species number described by taxonomists^49^, the value 22 was finally selected as the result of ABGD (Fig. 3).

**Figure 2:**
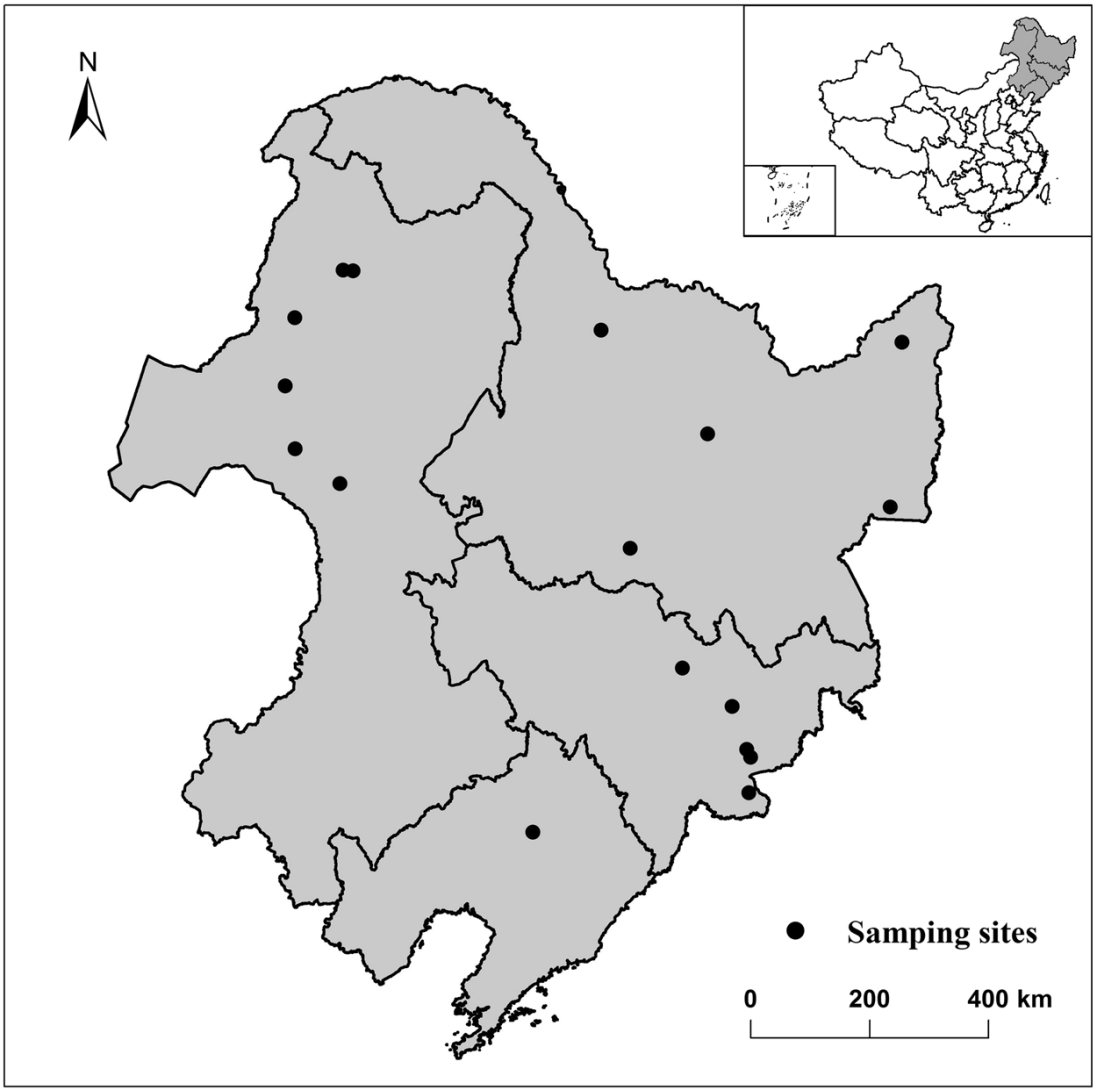
The distribution map of collection sites. The map is generated using software ArcGIS 10.3 (http://www.esri.com/software/arcgis) based on the geospatial data from the National Geomatics Center of China.

**Figure 3:**
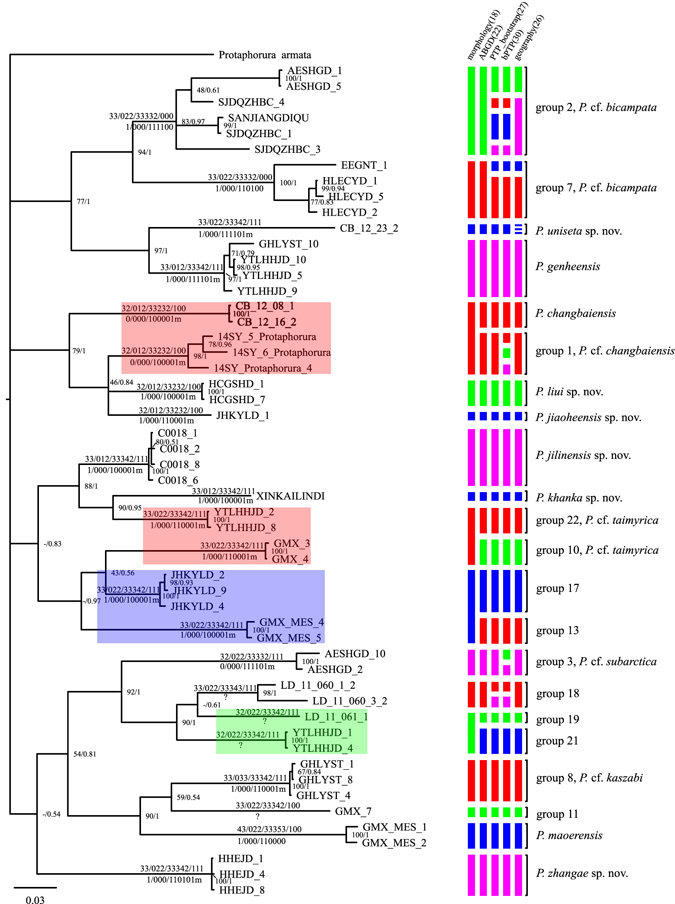
Summary from all species delimitation analyses on a Bayesian consensus tree. Node values represent likelihood bootstrap and posterior probabilities, respectively, with a–indicating nodes not compatible between the analyses. Colored bars represent hypothesized species groupings based on corresponding delimitation analyses. The geographical delimitation indicates the result of threshold of 50 kilometers. Colored clades on phylogeny are taxa morphologically unresolved. Number formulae above and below branches represent dorsal pseudocelli and ventral parapseudocelli, respectively.

For the single phylogenetic tree, bPTP.py estimated 26–40 (mean 30.98) putative species with a best supported partition of 30 species. With multiple bootstrap trees as input, 25–36 (mean 29.15) species were estimated by PTP.py, 27 in highest bootstrap supported partition. All clusters under both PTP.py and bPTP.py models formed monophyletic clades with high node bootstrap supports (>0.75, Fig. 3).

Under the primary hypothesis of 10 morphospecies, 27, 26, 26, 25, 20, 16, 16, 13 putative species were delimited using the distance threshold values 10, 20, 50, 100, 200, 300, 400, 500 kilometers respectively (Supplementary Tables S4 and S5). The estimates for number of species with different geographical threshold values showed an initial plateau around 26 and a second at 20 with a steep decline when distances between populations more than 100 kilometers. Because of the long-term persistence within restricted geographical limits for Collembola^60^, the plateau implied a sign of dispersal limits for species. Considering the possibility of no apparent gene flow across distances of tens of kilometers^60^, 26 putative species under a distance threshold of 50 kilometers were accepted for geographical criterion (Fig. 3).

The numbers of putative species estimated by the above methods were much greater than the ten morphospecies. Twenty-two MOTUs (GLC species), corresponding to ≈0.07 divergence threshold, were congruent across ABGD, PTP and geographical delimitations (Fig. 3). Among 22 putative species, the mean intra- and inter-specific distances were 0.93% (0–5.88%) and 15.91% (8.59–20.08%) for COI, respectively. Maximal divergences within species exceeded 5% in lineages group 2 (Gp 2) (Supplementary Tables S1 and S2, Supplementary Fig. S1).

When additional morphological characters were examined in combination with the traditional features, more morphological lineages were recognized, and most of them matched the 22-MOTUs partition. Among them, four twins cannot be distinguished on morphology: Gp 1 and Gp 5, Gp 10 and Gp 22, Gp 13 and Gp 17, Gp 19 and Gp 21 (Fig. 3). Finally, fourteen candidates were congruent across all three species validation criteria, including 2 known (Gp 9 and Gp 12), 6 new to science (Gp 4, Gp 6, Gp 14, Gp 15, Gp 16 and Gp 20), and the remaining which could not be designated a valid species name due to a lack of sufficient diagnostic information (e.g. Gp 11 and Gp 18 could not be further studied due to the lack of additional specimens; Gp 2, Gp 3, Gp 7 and Gp 8 have unclear taxonomical status due to the lack of detailed information available from similar type specimens from other collections) (Supplementary Tables S1–3). The morphological description of the six new species are given in the Supplementary files.

## Discussion

In the present study, we combined three types of delimitation data (morphology, molecular markers and geography) to resolve the taxonomical problems in the genus *Protaphorura*. The genus is most abundant and widely distributed group of Onychiuridae in northeast China^33^, and this gives us a good chance to collect a great many species and analyze both morphological and genetic information from fresh materials. Molecular markers, as well as geography, proved effective for delimitating. The results from the combination of morphological and molecular analyses, or morphological and geographical analyses were mostly congruent for species delimitation. In particular, morphology with ABGD provided the highest accuracy and could be used as a standard operational workflow in future taxonomy of the Onychiuridae.

Furthermore, the geographical distances may also help to discriminate taxa among closely related species, when the molecular information is unavailable. Geography criterion works well for species delimitation because of the low dispersal capabilities and long-term persistence^60, 61^, which result in high endemism and non-overlapping distribution. Geographical delimitation was mostly consistent with molecular approaches except delimitation for the widespread *P. uniseta* sp. nov. in the present study. Many collembolan species, which could be widely distributed in regions of large geographical scale, have been demonstrated to have cryptic diversity^62, 63^. Therefore, geographic criterion should be available for most onychiurids. We don’t encourage the single use of geographical distances to delimit species. Endemism extent and range of distributing area have not been estimated for most collembolan groups as well as most insects. As strategies implemented in this study, geographic criteria on the basis of preliminary morphological delimitation using simple morphological characters would be robust and accurate.

The inferences made here based on congruent results across several analyses are considered to be credible for species delimitation^37^. Several studies have combined the evidence from morphology and molecular markers in Collembola^55–58, 63, 64^. In our study, two new species delimitation methods based on molecular data (ABGD and PTP) were performed, and the results were mostly congruent. Coupled with the geographical evidence and using the Generalized Lineage Concept of species delimitation, the species of *Protaphorura* have been well discriminated here. Therefore, integrative taxonomy is essential for the accurate species identification of Onychiuridae, and it should be encouraged in the future taxonomical work.

Burkhardt’s work^59^ based on the results of molecular and morphological analyses supported Pomorski’s conclusion^27^, that the position of pseudocelli is more important than their number for taxonomy, especially for the ‘deformed’ pseudocelli which are reduced or supplementary pseudocelli present on only one side of the body. In a limited number of ‘deformed’ pseudocelli cases in our study (5 in 108 individuals), the diagnostic value of the pseudocelli formulae was validated in a number of species with the application of integrative taxonomy. Although frequent variations in the number and position of pseudocelli on the dorsal body have been reported in several species^26, 27^, we discovered here that the characters were relatively stable intraspecifically in the present 22 MOTUs which were both supported by the molecular and geographical evidence. Overall the results confirmed the importance of pseudocelli formulae for discriminating species in Onychiuridae^17, 18^ in the majority of cases.

Parapseudocelli, a long neglected structure in Onychiuridae, was first found by Rusek^28^ and never reported in other groups of Collembola. It is usually located on the ventral side of body, subcoxae 1 of legs and anal valves, while on the dorsal side of the body it occurs in some genera of the tribe *Hymenaphorurini* Pomorski, 1996. The parapseudocelli formula was first used as diagnostic character by Weiner^65^ and Pomorski^27, 66^. However, parapseudocelli are sometimes difficult to see and stated by several authors as “not seen” or “invisible” rather than “absent” in the descriptions. Therefore, the practicality of using this character in species diagnosis was questionable and it has not been widely used. In the present study, parapseudocelli formulae were shown to be stable and had no variations at the intraspecific level of *Protaphorura*. Variation in the number of the parapseudocelli is not common in other groups of Onychiuridae^12, 29, 67–69^ and for this reason they were proposed to be of great taxonomic values in species discrimination^70^. The instances of intraspecific variability reported for this character system before may correspond to inter-specific differences, because the variation reported referred to inter-populational rather than intra-populational comparisons^15, 32^. Compared to ten morphospecies recognized by traditional diagnostic characters, seven more have been recognized by the addition “parapseudocelli formulae” in our study, thus confirming views of Sun & Li^70^ on the diagnostic value of this character system.

While useful, several MOTUs, which were well separated by evidence from DNA sequences and geography, still could not been separated by morphological characters. Thus, other minor characters, which may have differences at the species level, were also explored. The number of chaetae on Th. sternum II, was often recorded in previous studies on Onychiuridae and can be relatively stable intraspecifically^13, 15, 29, 71, 72^, although it has not yet been treated as an important diagnostic character. When closely related taxa in this study are compared, the number of chaetae on Th. sternum II enabled G4 to be differentiated from Gp 20 (although this feature was variable in a small percentage of the examined specimens) and here we propose that it could be used as a main diagnostic character. The axial chaetae on abodominal terga IV–VI and the number of chaetae on subcoxa 1 of legs I–III, which are relatively stable at the intraspecific level in many genera of Onychiuridae^12–14, 29, 68, 72^, were also checked. However, they had a large ratio of intraspecific variations and could not be used as diagnostic characters in *Protaphorura*.

Despite the findings of this study, inadequacies in the morphological taxonomy of the *Protaphorura* still exist as evidenced by the presence of four pairs of morphologically cryptic species (e.g. Gp 1 and Gp 5, Gp 10 and Gp 22, Gp 13 and Gp 17, Gp 19 and Gp 21). This deficiency is caused by a lack of sufficient morphological characters or the existence of cryptic species, and requires further investigation.

## Material and Methods

### Taxon sampling

Samples were collected from areas of high biodiversity in northeast China, including the Changbai Mountain Range, the Greater and Lesser Khingan Range and the Sanjiang Plain (Fig. 2). Specimens were collected by Berlese extraction, and stored in 99% ethanol at −20 °C. One hundred and forty-four individuals from 17 populations were selected for molecular analysis. In this study, a population was defined as individuals collected from sites within a 5 kilometer radius of each other. The samples were stored pending analysis in the Key Laboratory of Wetland Ecology and Environment, Northeast Institute of Geography and Agroecology, Chinese Academy of Sciences, Changchun (NEIGA), China and the Department of Entomology, College of Plant Protection, Nanjing Agricultural University, Nanjing (NJAU), China.

### DNA extraction and sequencing

DNA was extracted using an Ezup Column Animal Genomic DNA Purification Kit (Sangon Biotech, Shanghai, China) following the manufacturer’s standard protocols. Specimens were kept in 75% ethanol for further morphological examination after the 1h-lysis buffer was transferred to the pipette containing silica column. Extractions were performed non-destructively (small tissue biopsy) allowing further morphological examination of the specimens. Primers were LCO1490/HCO2198 commonly used for metazoa^73^. Amplification volume and procedure were listed in Zhang *et al*.^63^. All PCR products were checked on a 1% agarose gel. Successful products were purified and sequenced in both directions by Majorbio (Shanghai, China) on the ABI 3730XL DNA Analyser (Applied Biosystems). Sequences were assembled in Sequencher 4.5 (Gene Codes Corporation, Ann Arbor, USA), and deposited in GenBank (Supplementary Table S3). Sequences were blasted in GenBank and checked for possible errors, then were preliminarily aligned using MAFFT v7.149 by the Q-INS-I strategy^74^. Alignments were checked and corrected manually, with a final 658 bp for COI.

### Phylogenetic analyses

Neighbour-joining (NJ) trees and genetic distances were calculated in MEGA 5.0^75^, with the Kimura-2 parameter model (K2P, Kimura 1980) and pairwise deletion for gaps. Node supports of NJ tree were evaluated through 1000 bootstrap replications.

Phylogenetic trees were reconstructed using maximum likelihood (ML) and Bayesian inference (BI) on online CIPRES services^76^. *Protaphorura armata* (Tullberg, 1869) from Europe was selected as the outgroup. To avoid the incorrect likelihood calculation, identical sequences were removed from the analyses. Best-fitting substitution models were assessed under the AIC and BIC criteria in jModelTest 2.1.7^77^, and the GTR + I + Γ model selected for subsequently analyses. ML trees were reconstructed in RAxML v8.2.4^78^ with the GTRGAMMAI model and 1000 bootstrap replicates. BI-analyses were conducted in MrBayes 3.2.6^79^. To avoid the problem of branch-length overestimation, the compound Dirichlet priors “brlenspr = unconstrained: gammadir (1, 1, 1, 1)” for branches lengths were incorporated^80^. The number of generations for the total analysis was set to 50 million, with the chain sampled every 5000 generations. The burn-in value was 25% and other parameters were set as default options. Evaluating effective sample size (ESS) values and state convergence were checked in Tracer 1.5^81^.

### Species delimitation

Species delimitation was performed using three data, morphology, mitochondrial marker COI, and geography. Using multiple approaches provided an acceptable error rate for species delimitation of 0.027^35^.

Morphology. The specimens for morphological examination were cleared in lactic acid, mounted in Marc André II solution and then studied using a Nikon Eclipse 80i microscope. Primary discrimination for morphospecies relied on the most traditionally important diagnostic characters among *Protaphorura* species including the formula and the positions of the pseudocelli on the dorsal body and subcoxa 1 of the legs, the presence or absence of additional microchaetae on abdominal terga I–III and V, and the relative position of the prespinal microchaetae on abdominal tergum VI. Other minor additional characters were carefully examined for assessing their potential values including the number and arrangement of parapseudocelli, the number of ventral mesothoracic chaetae, the axial chaetae on abdominal terga IV–VI, and the number of chaetae on subcoxa 1 of legs I–III. Specimens which displayed bilateral asymmetry in key diagnostic features were considered deformed and not included in our analysis.

Molecular approaches. Both distance-based (ABGD) and evolutionary model-based (PTP) methods were employed for the single mitochondrial marker COI without a priori species hypothesis. Prior intraspecific divergence varied from 0.001 (Pmin, a single nucleotide difference) to 0.14 (Pmax, threshold value applied in Collembola of Churchill^64^). Relative gap width was set to 1, with 20 recursive steps, 40 bids for graphic histogram of distances, K2P model for distance calculation and other parameters as default. An unrooted ML tree was generated in RAxML v8.2.4 with the GTRGAMMA model and 1000 bootstrap replicates. Identical sequences were removed and Bayesian PTP (bPTP) delimitation was performed on single unrooted tree in python script bPTP.py v0.51^50^. A total of 5 × 10^5^ generations were run with first 20% as burn-in. Maximum likelihood delimitation was also calculated in PTP.py 2.2 given 1000 trees derived from RAxML bootstrap analysis.

Geographical criterion. Because of the very low dispersal capabilities of springtails (except for some species which are in maritime islands and nunataks), vicariance and long-term persistence can occur within restricted geographical limits, even across distances of tens of kilometers^60, 61, 82^. Putative geographical species were delimited on the basis of primary hypotheses of morphospecies. For one morphospecies occurring in several geographical sites, populations, separated by distances greater than the hypothetical values (10–500 kilometers), were recognized as distinct putative species.

Species validation criteria. We used Generalized Lineage Concept (GLC) to determine the species boundary from our data. In this study, the operational criteria for final species recognition are conservative with trusted delimitations congruent across all analyses^37^: (i) monophyletic lineage (GLC) confirmed by phylogenetic inferences (it is also the a priori species hypothesis hidden in PTP); (ii) congruent across geographical and molecular delimitations; (iii) reliable morphological diagnosis with characters stably differing from other lineages. Lineages which met all of the above criteria were given a formal biological scientific name. Different lineages consistent across molecular and geographical analyses but of morphological stasis were considered cryptic species.

## Electronic supplementary material

Supplementary information
