# Supplementary material for: Delimiting species of Protaphorura (Collembola: Onychiuridae): integrative evidence based on morphology, DNA sequences and geography
Source: Sci Rep. 2017 Aug 15;7:8261. doi: 10.1038/s41598-017-08381-4 (PMC5557941; doi:10.1038/s41598-017-08381-4)
Supplement: Supplementary file 1 — Supplementary information [file 41598_2017_8381_MOESM1_ESM.pdf]

Supplementary information

Delimiting species of *Protaphorura* (Collembola: Onychiuridae): integrative evidence based on morphology, DNA sequences and geography

Xin Sun [1,3], Feng Zhang [2,4,*], Yinhuan Ding[2], Thomas W. Davies[5], Yu Li[3], Donghui Wu [1,*]
[1] Key Laboratory of Wetland Ecology and Environment, Northeast Institute of Geography and Agroecology, Chinese Academy of Sciences, Changchun 130102, China
[2] Department of Entomology, College of Plant Protection, Nanjing Agricultural University, Nanjing 210095, China
[3] Engineering Research Center of Chinese Ministry of Education for Edible and Medicinal Fungi, Jilin Agricultural University, Changchun 130118, China
[4] Key Laboratory of Zoological Systematics and Evolution, Institute of Zoology, Chinese Academy of Sciences, Beijing 100101, China
[5] Centre for Geography, Environment and Society, University of Exeter, Penryn, Cornwall TR10 9FE, UK
[*] Corresponding author; E-mail: xtmtd.zf@gmail.com, wudonghui@iga.ac.cn

This file includes:

Figure S1. Neighbour-joining tree based on COI for 144 specimens with node bootstrap values and species grouping shown.

Taxonomy. Description of the six new species.

Figure S2. *Protaphorura liui* Sun & Zhang sp. nov.. (a) Dorsal side of body. (b) Ventral side of head. (c) Ventral side of Abd. II–VI. Scales: 0.1 mm.

Figure S3. *Protaphorura liui* Sun & Zhang sp. nov.. (a) Distal part of leg. III. (b) Labium. (c) Postantennal organ, showing the sensory club. (d) Ant. III–IV. Scales: 0.1 mm (a, c, d), 0.01 mm (b).

Figure S4. *Protaphorura jiaoheensis* Sun & Zhang sp. nov.. (a) Dorsal side of body. (b) Labium. (c) Ventral side of head. (d) Ventral side of Abd. II–VI. Scales: 0.1 mm (a, c, d), 0.01 mm (b).

Figure S5. *Protaphorura jilinensis* Sun & Zhang sp. nov.. (a) Dorsal side of body. (b) Labium. (c) Postantennal organ. (d) Sensory organ of Ant. III. (e) Antenna. (f) Ventral side of Abd. I–VI. Scales: 0.1 mm (a, c, e, f), 0.01 mm (b, d).

Figure S6. *Protaphorura jilinensis* Sun & Zhang sp. nov.. (a) Ventral side of head. (b) Distal

part of leg. I. (c) Distal part of leg. III. (d) Th. sternum II. (e) Th. sternum III. (f) Central part of Abd. sternum IV. Scales: 0.1 mm (a, f), 0.01 mm (b–e).

Figure S7. *Protaphorura khanka* Sun & Zhang sp. nov.. (a) Dorsal side of body. (b) Ventral side of head. (c) Ventral side of Abd. II–VI. Scales: 0.1 mm.

Figure S8. *Protaphorura khanka* Sun & Zhang sp. nov.. (a) Anal valves. (b) Sensory organ of Ant. III. (c) Th. sternum II. (d) Central part of Abd. sternum IV. (e) Distal part of leg. III. Scales: 0.1 mm (a, d, e), 0.01 mm (b–c).

Figure S9. *Protaphorura uniseta* Sun & Zhang sp. nov.. (a) Dorsal side of body. (b) Ventral side of head. (c) Ventral side of Abd. II–VI. Scales: 0.1 mm.

Figure S10. *Protaphorura uniseta* Sun & Zhang sp. nov.. (a) Dorsal side of Abd. IV–VI. (b) Sensory organ of Ant. III. (c) Distal part of leg. III. Scales: 0.1 mm (a), 0.01 mm (b–c).

Figure S11. *Protaphorura zhangae* Sun & Zhang sp. nov.. (a) Dorsal side of body. (b) Sensory organ of Ant. III. (c) Labium. (d) Antenna. (e) Ventral side of Abd. I–VI. Scales: 0.1 mm (a, d, e), 0.01 mm (b–c).

Figure S12. *Protaphorura zhangae* Sun & Zhang sp. nov.. (a) Dorsal side of Abd. IV–VI. (b) Ventral side of head. Scales: 0.1 mm.

Table S1. Intraspecific divergence.

Table S2. Interspecific divergence.

Table S3. Molecular analysis information.

Table S4. Geographical distances between populations.

Table S5. The number of putative species delimited using different distance thresholds.

Table S6. The main diagnostic characters of *Protaphoura* from northeast China.

Table S7. Comparison and variation of the two new species having dorsal pso formula as 33/012/33342 and ventral psx as $1/000/100001^{m}$.

Figure S1. Neighbour-joining tree based on COI for 144 specimens with node bootstrap values and species grouping shown.

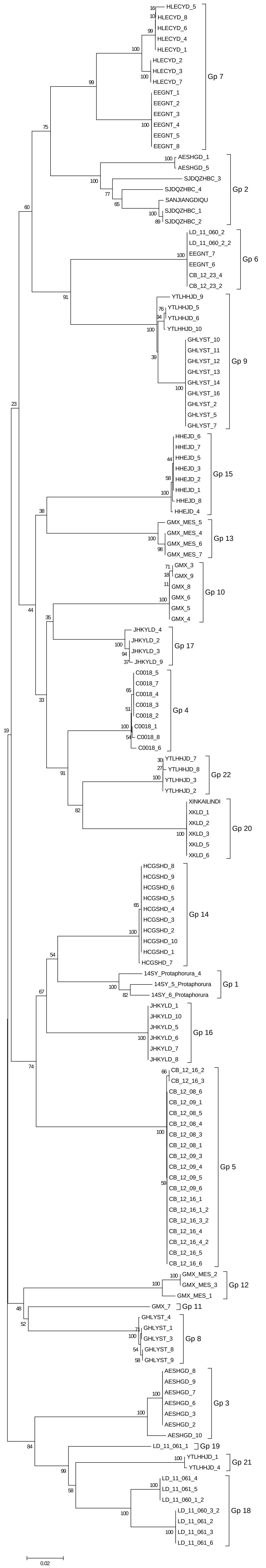

Gp 7
Gp 2
Gp 6
Gp 9
Gp 15
Gp 13
Gp 10
Gp 17
Gp 4
Gp 22
Gp 20
Gp 14
Gp 1
Gp 16
Gp 5
Gp 12
Gp 11
Gp 8
Gp 3
Gp 19
Gp 21
Gp 18
0.02

Taxonomy

Taxonomical description methods:

Labial type is named after Fjellberg[83]. Labial areas and chaetal nomenclature follow Massoud[84] and D'Haese[85]. Chaetae on anal valves are named following Yoshii[86]. Chaetae on the furcal area are classified in accordance with Weiner[87]. The formulae of pseudocelli, parapseudocelli and pseudopores are the number of pseudocelli, parapseudocelli or pseudopores by half-tergum (dorsally) or half-sternum (ventrally) as follows: head anterior, head posterior/Th. I, Th. II, Th. III/Abd. I, Abd. II, Abd. III, Abd. IV, Abd. V (for instance: 32/012/33232). The tibiotarsus chaetotaxy formula follows Deharveng[88] and is expressed as: total number of chaetae and also number of chaetae in whorl A+T, B and basal chaetae, for example: 22 (11, 8, 3).

Abbreviations used in descriptions. Ant.—antennal segments, PAO—postantennal organ, Th.—thoracic segments, Abd.—abdominal segments, ms—microsensillum, pso—pseudocellus, psx—parapseudocellus, psp—pseudopore, ^{m}—unpaired parapseudocellus and pseudopore, s—sensory chaeta on the body, p-chaeta—chaeta of row p, M chaeta—submedial posterior macrochaeta on Abd. tergum V, sp—posterior s-chaeta on Abd. tergum V, AS—anal spines, s'—additional microchaeta on Abd. terga I–III and V, set above the submedial pso.

***Protaphorura liui* Sun & Zhang sp. nov.**

(Supplementary Figures S2, S3; Supplementary Table S6)

Type material. Holotype female, paratypes 9 females, 1 male and 20 juveniles on slides. China: Heilongjiang Province: Jiamusi City: Hanconggou town (43.07765° N, 128.01729° E), litter and soil, Berlese extraction, 28.IX.2014, leg. Wu Donghui et al.

Description. Body color white in alcohol. Size 1.40–1.55 mm in females, 1.30 mm in male; holotype 1.55 mm. Body slender and elongated.

Pseudocellar formula dorsally: 32/012/33232, ventrally: 10/000/0000 (Fig. S2a–c); subcoxa 1 of legs I–III with 1, 0, 0 pso respectively (Fig. S2a). Parapseudocellar formula ventrally: 1/000/100001^{m} (Fig. S2b–c); subcoxa 1 of legs I–III with 1, 1, 1 psx respectively (Fig. S2a). Pseudopores formula dorsally: 00/011/1111, ventrally: 0/111/01^{m}1^{m}1^{m} (Fig. S2a–c).

Head. Antennae nearly as long as head. Ant. IV with subapical organite; basolateral ms above the second proximal row of chaetae (Fig. S3d). Ant. III sensory organ composed of 5 papillae, 5 guard chaetae, 2 small sensory rods, 2 granulated sensory clubs and a lateral ms (Fig. S3d). Ant. II with 17 chaetae. Ant. I with 11 chaetae. Antennal bases with distinct granulation.

PAO composed of 26–36 simple vesicles (Fig. S3c). 4+4 p-chaetae between postero-

internal pso on head (Fig. S2a). Mandible with strong molar plate and 4 apical teeth. Maxilla bearing 3 teeth and 6 lamellae. Maxillary palp simple with 1 basal chaeta and 2 sublobal hairs. Labral chaetae formula 4/342. Labium with 7 proximal, 4 basomedian (E, F, G and f) and 6 basolateral chaetae (a, b, c, d, e, e'); labial type A, papillae A–E respectively with 1, 4, 0, 3, 3 guard chaetae (Fig. S3b). Postlabial chaetae 5+5 along ventral groove (Fig. S2b).

Body chaetotaxy. Dorsal chaetotaxy usually with some asymmetry, well differentiated into macro-, meso- and microchaetae. Chaetae s very weakly marked. Th. terga II–III with ms laterally (Fig. S2a). Th. tergum I with 11+11 chaetae (Fig. S2a). Abd. terga I–III and V without s' (Fig. S2a). Abd. tergum V with p0 axial chaetae, Abd. tergum VI with m0 axial chaeta (Fig. S2a). Ratio M/sp on Abd. V as 2, M/AS as 3 (Fig. S2a). Straight lines passing through the bases of chaetae a1 and m1 parallel on Abd. tergum VI (Fig. S2a). Th. sterna I–III with 1+1, 1+1, 2+2 chaetae respectively. Ventral tube with 8–10+8–10 distal chaetae and 2+2 basal chaetae, without anterior chaetae. Furca reduced to cuticular pocket with 2+2 dental chaetae—1+1 chaetae located on a cuticular fold, remaining 1+1 chaetae located distinctly below fold; three manubrial rows of chaetae present posteriorly to dental chaetae (Fig. S2c). Female genital plate with 17–20 chaetae, male genital chaetae plate with 42 chaetae. Male ventral organ absent. Anal valves with numerous acuminate chaetae; each lateral valve with a0, 2a1 and 2a2; upper valve with chaetae a0, 2a1, 2b1, 2b2, c0, 2c1, 2c2 (Fig. S2c).

Appendages. Subcoxa 1 of legs I–III with 5, 5 and 5 chaetae, subcoxa 2 with 1, 5 and 5 chaetae, respectively. Tibiotarsi of legs I–III with 22 (11, 8, 3), 23 (11, 8, 4) and 23 (11, 8, 4) chaetae, respectively (Fig. S3a). Unguis with small inner denticle. Unguiculus slender and pointed, 1.1 times as long as inner edge of unguis, without inner basal lamella (Fig. S3a). Anal spines set on distinct papillae, about 0.6–0.7 times as long as inner edge of unguis (Fig. S3a).

Etymology. The name of the new species refers to the name of the husband of the first author Liu Yunda.

Ecology. Found in a broad-leaved forest of the town.

Remarks. *Protaphorura liui* sp. nov., *P. changbaiensis* Sun, Zhang & Wu, 2013 and *P. jiaoheensis* sp. nov. (see below) are very similar to each other in the main morphological characters and can be classed to a group with dorsal pso formula as 32/012/33232. However, they are separated easily from each other by the mean genetic distance (0.134 between *P. changbaiensis* and *P. jiaoheensis* sp. nov., 0.130 between *P. changbaiensis* and *P. liui* sp. nov., 0.115 between *P. jiaoheensis* sp. nov. and *P. liui* sp. nov.) (Table S2). Morphologically, they can also be separated by the ventral psx formula (*P. changbaiensis* as $0/000/100001^{m}$ (psx on upper valve is ignored in the original description), *P. liui* sp. nov. as $1/000/100001^{m}$, *P. jiaoheensis* sp.

nov. as 1/000/110001^m) (Table S6).

The three-species group has the same pso formula of Abd. I–V terga (33232) as the Crimean species *P. ajudagi* Pomorski, Skarżyński & Kaprus', 1998 and the Mongolian species *P. microcellata* (Dunger, 1978). However, the three-species group differs from *P. ajudagi* by the absence of inner pso on Th. II tergum (present in *P. ajudagi*) and the posterior pso ventrally on head (present in *P. ajudagi*), the shape of sensory clubs on Ant. III sensory organ (smooth in *P. ajudagi*), the presence of chaetae on Th. I–III sterna (absent in *P. ajudagi*) and the absence of the male ventral organ (present in *P. ajudagi*); it can be also distinguished easily from *P. microcellata* by absence of inner pso on Th. II tergum (present in *P. microcellata*) and the presence of pso on subcoxa 1 of leg I (absent in *P. microcellata*).

***Protaphorura jiaoheensis* Sun & Zhang sp. nov.**

(Supplementary Figure S4; Supplementary Table S6)

Type material. Holotype female, paratypes 9 females, 7 males and 2 juveniles on slides. China: Jilin Province: Jiaohe City (43.80567° N, 127.17435° E), litter and soil, Berlese extraction, 28.IX.2014, leg. Wu Donghui *et al.*

Description. Body color white in alcohol. Size 1.60–1.75 mm in females, 1.40–1.50 mm in males; holotype 1.60 mm. Body slender and elongated.

Pseudocellar formula dorsally: 32/012/33232, ventrally: 10/000/0000 (Fig. S4a, c, d); subcoxa 1 of legs I–III with 1, 0, 0 pso respectively (Fig. S4a). Parapseudocellar formula ventrally: 1/000/110001^m (Fig. S4c, d); subcoxa 1 of legs I–III with 1, 1, 1 psx respectively. Pseudopores formula dorsally: 00/011/1111, ventrally: 0/111/01^{m}1^{m}1^m (Fig. S4a, c, d).

Head. Antennae nearly as long as head. Ant. IV with a subapical organite; basolateral ms above the second proximal row of chaetae. Ant. III sensory organ composed of 5 papillae, 5 guard chaetae, 2 small sensory rods, 2 granulated sensory clubs and a lateral ms. Ant. II with 17 chaetae. Ant. I with 11 chaetae. Antennal bases with distinct granulation.

PAO composed of 26–30 simple vesicles (Fig. S4a). 4+4 p-chaetae between postero-internal pso on head (Fig. S4a). Mandible with strong molar plate and 4 apical teeth. Maxilla bearing 3 teeth and 6 lamellae. Maxillary palp simple with 1 basal chaeta and 2 sublobal hairs. Labral chaetae formula 4/342. Labium with 7 proximal, 4 basomedian (E, F, G and f) and 6 basolateral chaetae (a, b, c, d, e, e'); labial type A, papillae A–E respectively with 1, 4, 0, 3, 3 guard chaetae (Fig. S4b). Postlabial chaetae 5+5 along ventral groove (Fig. S4c).

Body chaetotaxy. Dorsal chaetotaxy usually with some asymmetry, well differentiated into macro-, meso- and microchaetae. Chaetae s very weakly marked. Th. terga II–III with ms

laterally (Fig. S4a). Th. tergum I with 1011+10–11 chaetae (Fig. S4a). Abd. terga I–III and V without s' (Fig. S4a). Abd. tergum V with p0 axial chaetae, Abd. tergum VI with m0 axial chaeta (Fig. S4a). Ratio M/sp on Abd. V as 2, M/AS as 3 (Fig. S4a). Straight lines passing through the bases of chaetae a1 and m1 parallel on Abd. tergum VI (Fig. S4a). Th. sterna I–III with 1+1, 1+1, 2+2 chaetae respectively. Ventral tube with 10+10 distal chaetae and 2+2 basal chaetae, without anterior chaetae. Furca reduced to cuticular pocket with 2+2 dental chaetae—1+1 chaetae located on a cuticular fold, remaining 1+1 chaetae located distinctly below fold; three manubrial rows of chaetae present posteriorly to dental chaetae (Fig. S4d). Female genital plate with 18–20 chaetae, male genital chaetae plate with 42–44 chaetae. Male ventral organ absent. Anal valves with numerous acuminate chaetae; each lateral valve with a0, 2a1 and 2a2; upper valve with chaetae a0, 2a1, 2b1, 2b2, c0, 2c1, 2c2 (Fig. S4d).

Appendages. Subcoxa 1 of legs I–III with 5, 5 and 5 chaetae, subcoxa 2 with 1, 5 and 5 chaetae, respectively. Tibiotarsi of legs I–III with 22 (11, 8, 3), 23 (11, 8, 4) and 23 (11, 8, 4) chaetae, respectively. Unguis with small inner denticle. Unguiculus slender and pointed, 1.1 times as long as inner edge of unguis, without inner basal lamella. Anal spines set on distinct papillae, about 0.6 times as long as inner edge of unguis (Fig. S4a).

Etymology. The name of the new species refers to locality where it was collected.

Ecology. Found in a broad-leaved forest of the city.

Remarks. See above remarks on *Protaphorura liui* sp. nov..

Protaphorura jilinensis **Sun & Zhang sp. nov.**

(Supplementary Figures S5, S6; Supplementary Table S3)

Type material. Holotype female, paratypes 14 females and 5 males on slides. China: Jilin Province: Changbai Mountain (alt. ca 1700 m, 41.76° N, 127.94° E), litter and soil, Berlese extraction, 15.VIII.2009, leg. Wu Donghui.

Description. Body color white in alcohol. Size 2.0–2.2 mm in females, 1.8–1.9 mm in males; holotype 2.0 mm. Body slender and elongated.

Pseudocellar formula dorsally: 33/012/33342, ventrally: 10/000/0000 (Figs S5a, f, S6a); subcoxa 1 of legs I–III with 1, 1, 1 pso respectively (Fig. S5a). Parapseudocellar formula ventrally: 1/000/100001^{m} (Figs S5f, S6a); subcoxa 1 of legs I–III with 1, 1, 1 psx respectively (Fig. S5a). Pseudopores formula dorsally: 00/011/1111, ventrally: 0/111/01^{m}1^{m}1^{m} (Figs S5a, f, S6a).

Head. Antennae nearly as long as head. Ant. IV with a subapical organite; basolateral ms above the second proximal row of chaetae. Ant. III sensory organ composed of 5 papillae, 5

guard chaetae, 2 small sensory rods, 2 granulated sensory clubs and a lateral ms (Fig. S5d–e). Ant. II with 18 chaetae. Ant. I with 11 chaetae. Antennal bases with distinct granulation. PAO composed of 30–40 simple vesicles (Fig. S5c). 4+4 p-chaetae between postero-internal pso on head (Fig. S5a). Mandible with strong molar plate and 4 apical teeth. Maxilla bearing 3 teeth and 6 lamellae. Maxillary palp simple with 1 basal chaeta and 2 sublobal hairs. Labral chaetae formula 4/342. Labium with 7 proximal, 4 basomedian (E, F, G and f) and 6 basolateral chaetae (a, b, c, d, e, e'); labial type A, papillae A–E respectively with 1, 4, 0, 3, 3 guard chaetae (Fig. S5b). Postlabial chaetae 5+5 along ventral groove (Fig. S6a).

Body chaetotaxy. Dorsal chaetotaxy usually with some asymmetry, well differentiated into macro-, meso- and microchaetae. Chaetae s very weakly marked. Th. terga II–III with ms laterally (Fig. S5a). Th. tergum I with 12–13+12–13 chaetae (Fig. S5a). Abd. terga I–III and V without s' (Fig. S5a). Abd. terga IV–V with several asymmetrical chaetae along the axial line, Abd. tergum IV with one or two m0 axial chaetae, Abd. tergum V with p0 axial chaeta, m0 present or not, Abd. tergum VI with m0 axial chaeta (Fig. S5a). Ratio M/sp on Abd. V as 1.4–1.5, M/AS as 3.2–3.5 (Fig. S5a). Straight lines passing through the bases of chaetae a1 and m1 parallel on Abd. tergum VI (Fig. S5a). Th. sterna I–III with 1+1, 1+1, 2+2 chaetae respectively (Fig. S6d–e), rarely 1+2 on Th. sternum II. Ventral tube with 8–11+8–11 distal chaetae and 2+2 basal chaetae, without anterior chaetae (Fig. S5f). Furca reduced to cuticular pocket with 2+2 dental chaetae—1+1 chaetae located on a cuticular fold, remaining 1+1 chaetae located distinctly below fold; three manubrial rows of chaetae present posteriorly to dental chaetae (Figs S5f, S6f). Female genital plate with 20–24 chaetae, male genital chaetae plate with 36–48 chaetae. Male ventral organ absent. Anal valves with numerous acuminate chaetae; each lateral valve with a0, 2a1 and 2a2; upper valve with chaetae a0, 2a1, 2b1, 2b2, c0, 2c1, 2c2 (Fig. S5f).

Appendages. Subcoxa 1 of legs I–III with 5, 7 and 5(6, 7) chaetae, subcoxa 2 with 1, 5 and 5 chaetae, respectively. Tibiotarsi of legs I–III with 22 (11, 8, 3), 24 (11, 8, 5) and 23 (11, 8, 4) chaetae, respectively (Fig. S6c–d). Unguis with small inner denticle. Unguiculus slender and pointed, as long as inner edge of unguis, without inner basal lamella (Fig. S6c–d). Anal spines set on distinct papillae, about 0.6–0.7 times as long as inner edge of unguis (Fig. S5a).

Etymology. The name of the new species refers to name of the province where it was collected.

Ecology. Found in coniferous forest.

Remarks. *Protaphorura jilinensis* sp. nov. is very similar to *P. khanka* sp. nov. (see below) in most morphological characters, but they are separated easily from each other by the mean

0.106 genetic distance (Table S2). After re-examination of fresh and voucher specimens, we found several minor differences between the two species as shown in Table S7, although these characters usually show variations in species.

The two new species have the same dorsal pseudocellar formula (33/012/33342) with the species *P. dzherga* Gulgenova & Potapov, 2013 which is described from Russia. However, the two new species differ from *P. dzherga* by the numbers of pso on subcoxa 1 of legs I–III (1,1,1 in the two new species, absent in *P. dzherga*), the ventral psx formula (1/000/100001^{m} in the two new species, 0/000/11000 in *P. dzherga*), and the number of papillae in Ant. III sensory organ (5 in the two new species, 4 in *P. dzherga*).

Protaphorura khanka **Sun & Zhang sp. nov.**

(Supplementary Figures S7, S8; Supplementary Table S6)

Type material. Holotype female, paratypes 14 females and 5 males on slides. China: Heilongjiang Province: Lake Khanka (45.373181° N, 132.332138° E), litter and soil, Berlese extraction, 18.IX.2011, leg. Wu Haitao and Song Lihong.

Description. Body color white in alcohol. Size 1.50–1.75 mm in females, 1.30–1.45 mm in males; holotype 1.60 mm. Body slender and elongated.

Pseudocellar formula dorsally: 33/012/33342, ventrally: 10/000/0000 (Fig. S7a–c); subcoxa 1 of legs I–III with 1, 1, 1 pso respectively (Fig. S7a). Parapseudocellar formula ventrally: 1/000/100001^{m} (Fig. S7b–c); subcoxa 1 of legs I–III with 1, 1, 1 psx respectively (Fig. S7a). Pseudopores formula dorsally: 00/011/1111, ventrally: 0/111/01^{m}1^{m}1^{m} (Fig. S7a–c).

Head. Antennae nearly as long as head. Ant. IV with a subapical organite; basolateral ms above the second proximal row of chaetae. Ant. III sensory organ composed of 5 papillae, 5 guard chaetae, 2 small sensory rods, 2 granulated sensory clubs and a lateral ms (Fig. S8b). Ant. II with 18 chaetae. Ant. I with 11 chaetae. Antennal bases with distinct granulation.

PAO composed of 30–36 simple vesicles. 4+4 p-chaetae between postero-internal pso on head (Fig. S7a). Mandible with strong molar plate and 4 apical teeth. Maxilla bearing 3 teeth and 6 lamellae. Maxillary palp simple with 1 basal chaeta and 2 sublobal hairs. Labral chaetae formula 4/342. Labium with 7 proximal, 4 basomedian (E, F, G and f) and 6 basolateral chaetae (a, b, c, d, e, e'); labial type A, papillae A–E respectively with 1, 4, 0, 3, 3 guard chaetae. Postlabial chaetae 5–6+5–6 along ventral groove (Fig. S7b).

Body chaetotaxy. Dorsal chaetotaxy usually with some asymmetry, well differentiated into macro-, meso- and microchaetae. Chaetae s very weakly marked. Th. terga II–III with ms

laterally (Fig. S7a). Th. tergum I with 12–13+12–13 chaetae (Fig. S7a). Abd. terga I–III and V without s' (Fig. S7a). Abd. terga IV–V with several asymmetrical chaetae along the axial line, Abd. tergum IV with one or two m0 axial chaetae, Abd. tergum V with p0 axial chaeta, m0 present or not, Abd. tergum VI with m0 axial chaeta (Fig. S7a). Ratio M/sp on Abd. V as 1.5–1.6, M/AS as 3–3.5 (Fig. S7a). Straight lines passing through the bases of chaetae a1 and m1 parallel on Abd. tergum VI (Fig. S7a). Th. sterna I–III with 1+1, 2+2, 2+2 chaetae respectively (Fig. S8c), rarely 1+2 or 1+1 on Th. sternum II. Ventral tube with 10–13+10–13 distal chaetae and 2+2 basal chaetae, without anterior chaetae. Furca reduced to cuticular pocket with 2+2 dental chaetae—1+1 chaetae located on a cuticular fold, remaining 1+1 chaetae located distinctly below fold; three manubrial rows of chaetae present posteriorly to dental chaetae (Fig. S8d). Female genital plate with 22–26 chaetae, male genital chaetae plate with 28–34 chaetae. Male ventral organ absent. Anal valves with numerous acuminate chaetae; each lateral valve with a0, 2a1 and 2a2; upper valve with chaetae a0, 2a1, 2b1, 2b2, c0, 2c1, 2c2 (Fig. S8a).

Appendages. Subcoxa 1 of legs I–III with 5, 7(6) and 5 chaetae, subcoxa 2 with 1, 5 and 5 chaetae, respectively. Tibiotarsi of legs I–III with 22–23 (11, 8, 4(3)), 24 (11, 8, 5) and 23 (11, 8, 4) chaetae, respectively (Fig. S8e). Unguis with small inner denticle. Unguiculus slender and pointed, as long as inner edge of unguis, without inner basal lamella (Fig. S8e). Anal spines set on distinct papillae, about 0.6 times as long as inner edge of unguis (Fig. S7a).

Etymology. The name of the new species refers to locality where it was collected.

Ecology. Found in coniferous and broad-leaved mixed forest.

Remarks. See above remarks on *Protaphorura jilinensis* sp. nov.

***Protaphorura uniseta* Sun & Zhang sp. nov.**

(Supplementary Figures S9, S10; Supplementary Table S6)

Type material. Holotype female, paratypes 1 female and 2 males on slides. China: Heilongjiang Province: Lesser Khingan Tree Farm (49.058176° N, 127.074391° E), litter, Berlese extraction, 1.VII.2011, leg. Sun Xin et al.

Other material. 2 females on slides. China: Inner Mongolia: Hulun Buir: Ergun City (50.12214° N, 120.20458° E), soil, Berlese extraction, 23. IX. 2014, leg. Li Jiujia.

Description. Body color white in alcohol. Size 2.1–2.2 mm in females, 1.6–1.7 mm in males; holotype 2.1 mm. Body slender and elongated.

Pseudocellar formula dorsally: 33/022/33342, ventrally: 10/000/0000 (Fig. S9a–c); subcoxa 1 of legs I–III with 1, 1, 1 pso respectively (Fig. S9a). Parapseudocellar formula ventrally: 1/000/111101^{m} (Fig. S9c); subcoxa 1 of legs I–III with 1, 1, 1 psx respectively (Fig.

S9a). Pseudopores formula dorsally: 00/011/1111, ventrally: 0/111/01^{m}1^{m}1^{m} (Fig. S9a–c).

Head. Antennae nearly as long as head. Ant. IV with a subapical organite; basolateral ms above the second proximal row of chaetae. Ant. III sensory organ composed of 5 papillae, 5 guard chaetae, 2 small sensory rods, 2 granulated sensory clubs and a lateral ms (Fig. S10b). Ant. II with 16–17 chaetae. Ant. I with 11 chaetae. Antennal bases with distinct granulation.

PAO composed of 34–40 simple vesicles. 4+4 p-chaetae between postero-internal pso on head (Fig. S9a). Mandible with strong molar plate and 4 apical teeth. Maxilla bearing 3 teeth and 6 lamellae. Maxillary palp simple with 1 basal chaeta and 2 sublobal hairs. Labral chaetae formula 4/342. Labium with 7 proximal, 4 basomedian (E, F, G and f) and 6 basolateral chaetae (a, b, c, d, e, e') (Fig. S9b); labial type A, papillae A–E respectively with 1, 4, 0, 3, 3 guard chaetae. Postlabial chaetae 5+5 along ventral groove (Fig. S9b).

Body chaetotaxy. Dorsal chaetotaxy usually with some asymmetry, well differentiated into macro-, meso- and microchaetae. Chaetae s very weakly marked. Th. terga II–III with ms laterally (Fig. S9a). Th. tergum I with 12–14+12–14 chaetae (Fig. S9a). Abd. terga I–III and V without s' (Fig. S9a). Abd. terga IV–V with several asymmetrical chaetae along the axial line, Abd. tergum IV with one or two m0 axial chaetae, Abd. tergum V with m0 and p0 axial chaetae, Abd. tergum VI with m0 axial chaeta (Figs S9a, S10a). Ratio M/sp on Abd. V as 1.4, M/AS as 2.5 (Figs S9a, S10a). Straight lines passing through the bases of chaetae a1 and m1 parallel on Abd. tergum VI (Figs S9a, S10a). Th. sterna I–III with 1+1, 1+1, 2+2 chaetae respectively. Ventral tube with 9–10+9–10 distal chaetae and 2+2 basal chaetae, without anterior chaetae. Furca reduced to cuticular pocket with 2+2 dental chaetae—1+1 chaetae located on a cuticular fold, remaining 1+1 chaetae located distinctly below fold; three manubrial rows of chaetae present posteriorly to dental chaetae (Fig. S9c). Female genital plate with 25 chaetae, male genital chaetae plate with 34–40 chaetae. Male ventral organ absent. Anal valves with numerous acuminate chaetae; each lateral valve with a0, 2a1 and 2a2; upper valve with chaetae a0, 2a1, 2b1, 2b2, c0, 2c1, 2c2 (Fig. S9c).

Appendages. Subcoxa 1 of legs I–III with 6, 6–7 and 5–6 chaetae, subcoxa 2 with 1, 5 and 5 chaetae, respectively. Tibiotarsi of legs I–III with 22 (11, 8, 3), 24 (11, 8, 5) and 23 (11, 8, 4) chaetae, respectively (Fig. S10c). Unguis with small inner denticle. Unguiculus slender and pointed, as long as inner edge of unguis, without inner basal lamella (Fig. S10c). Anal spines set on distinct papillae, about 0.6–0.7 times as long as inner edge of unguis (Fig. S9a).

Etymology. The name of the new species refers to having only one ventral mesothoracic chaeta on each side.

Ecology. Found in broad-leaved forest.

Remarks. *Protaphorura uniseta* sp. nov. and *P. zhangae* sp. nov. (see below) belong to the species group which has the dorsal pso formula 33/022/33342 (Sun *et al.*, 2015). *P. uniseta* sp. nov. is most similar to *P. boedvarssoni* Pomorski, 1993 in having the same psx formula; but they can be recognized easily by the number of ventral mesothoractic chaetae (1+1 in the new species and 2+2 in *P. boedvarssoni*) and the presence of chaeta s' on Abd. terga I–III (absent in the new species and present in *P. boedvarssoni*).

Protaphorura zhangae **Sun & Zhang sp. nov.**

(Supplementary Figures S11, S12; Supplementary Table S6)

Type material. Holotype female, paratypes 4 females and 9 males on slides. China: Inner Mongolia: Honghuaerji City (48.13709° N, 119.76073° E), litter, Berlese extraction, 22.IX.2014, leg. Li Jiujia.

Description. Body color white in alcohol. Size 1.78–1.80 mm in females, 1.5–1.7 mm in males; holotype 1.80 mm. Body slender and elongated.

Pseudocellar formula dorsally: 33/022/33342, ventrally: 10/000/0000 (Figs S11a, e, S12b); subcoxa 1 of legs I–III with 1, 1, 1 pso respectively (Fig. S11a). Parapseudocellar formula ventrally: 1/000/110101^{m} (Figs S11e, S12b); subcoxa 1 of legs I–III with 1, 1, 1 psx respectively (Fig. S11a). Pseudopores formula dorsally: 00/011/1111, ventrally: 0/111/$01^{m}1^{m}1^{m}$ (Figs S11a, e, S12b).

Head. Antennae nearly as long as head. Ant. IV with a subapical organite; basolateral ms above the second proximal row of chaetae. Ant. III sensory organ composed of 5 papillae, 5 guard chaetae, 2 small sensory rods, 2 granulated sensory clubs and a lateral ms (Fig. S11b). Ant. II with 17 chaetae. Ant. I with 11 chaetae. Antennal bases with distinct granulation.

PAO composed of 24–30 simple vesicles. 4+4 p-chaetae between postero-internal pso on head (Fig. S11a). Mandible with strong molar plate and 4 apical teeth. Maxilla bearing 3 teeth and 6 lamellae. Maxillary palp simple with 1 basal chaeta and 2 sublobal hairs. Labral chaetae formula 4/342. Labium with 7 proximal, 4 basomedian (E, F, G and f) and 6 basolateral chaetae (a, b, c, d, e, e') (Fig. S12b); labial type A, papillae A–E respectively with 1, 4, 0, 3, 3 guard chaetae (Fig. S11c). Postlabial chaetae 5+5 along ventral groove (Fig. S12b).

Body chaetotaxy. Dorsal chaetotaxy usually with some asymmetry, well differentiated into macro-, meso- and microchaetae. Chaetae s very weakly marked. Th. terga II–III with ms laterally (Fig. S11a). Th. tergum I with 12+12 chaetae (Fig. S11a). Abd. terga I–III and V without s' (Figs S11a, S12a). Abd. terga IV–V with several asymmetrical chaetae along the axial line, Abd. tergum IV with m0 axial chaeta, Abd. tergum V with m0 and p0 axial chaetae,

Abd. tergum VI with m0 axial chaeta (Figs S11a, S12a). Ratio M/sp on Abd. V as 1.8–2.0, M/AS as 3 (Figs S11a, S12a). Straight lines passing through the bases of chaetae a1 and m1 parallel on Abd. tergum VI (Figs S11a, S12a). Th. sterna I–III with 1+1, 1+1, 2+2 chaetae respectively. Ventral tube with 11–12+11–12 distal chaetae and 2+2 basal chaetae, without anterior chaetae. Furca reduced to cuticular pocket with 2+2 dental chaetae—1+1 chaetae located on a cuticular fold, remaining 1+1 chaetae located distinctly below fold; three manubrial rows of chaetae present posteriorly to dental chaetae (Fig. S11e). Female genital plate with 20–21 chaetae, male genital chaetae plate with 48 chaetae. Male ventral organ absent. Anal valves with numerous acuminate chaetae; each lateral valve with a0, 2a1 and 2a2; upper valve with chaetae a0, 2a1, 2b1, 2b2, c0, 2c1, 2c2 (Fig. S11e).

Appendages. Subcoxa 1 of legs I–III with 5, 5 and 5 chaetae, subcoxa 2 with 1, 5 and 5 chaetae, respectively. Tibiotarsi of legs I–III with 23 (11, 8, 4), 24 (11, 8, 5) and 23 (11, 8, 4) chaetae, respectively. Unguis with small inner denticle. Unguiculus slender and pointed, as long as inner edge of unguis, without inner basal lamella. Anal spines set on distinct papillae, about 0.6 times as long as inner edge of unguis (Fig. S11a).

Etymology. The name of the new species is in memory of Professor Zhang Xueping who was an excellent ecologist from Harbin Normal University.

Ecology. Found in broad-leaved forest.

Remarks. *P. zhangae* sp. nov. belongs to the species group which has the dorsal pso formula 33/022/33342 (Sun *et al.*, 2015). However, *P. zhangae* sp. nov. differs from *P. uniseta* sp. nov., *P. taimyrica* (Martynova, 1976), *P. boedvarssoni* Pomorski, 1993 and *P. stogovi* Pomorski, 1993 as having different psx formula (1/000/110101^{m} in *P. zhangae* sp. nov., 1/000/111101^{m} in *P. uniseta* sp. nov. and *P. boedvarssoni*, 1/000/110001^{m} in *P. taimyrica* and *P. stogovi*); it can also be separated from *P. montana* (Mateos & Arbea, 1986), *P. paranemorata* (Selga, 1962), *P. quercetana* (Mateos & Arbea, 1986), *P. spinoidea* (Steiner, 1955), *P. subnemorata* (Gisin, 1957) and *P. zori* (Martynova, 1975) by the relative position of prespinal microchaetae on Abd. tergum VI (parallel in *P. zhangae* sp. nov. and convergent in the latter ones), and from *P. sensillata* (Khanislamova, 1986) by the presence of chaeta s' on Abd. tergum V (absent in *P. zhangae* sp. nov. and present in *P. sensillata*).

References

1. Fjellberg, A. The labial palp in Collembola. *Zool. Anz.* **237**, 309–330 (1999).
2. Massoud, Z. Monographie des Neanuridae, Collemboles Poduromorphes à pièces buccales modifiées. *Biol. Am. Australe* **3**, 7–399 (1967).

3. D’Haese, C. A. Homology and morphology in Poduromorpha (Hexapoda, Collembola). *Eur. J. Entomol.* **101**, 385–407 (2003).

4. Yoshii, R. Identity of some Japanese Collembola IV. “*Deuteraphorura*” group of *Onychiurus* - continued. *Ann. Speleol. Res. Inst. Japan* **14**, 1–15 (1996).

5. Weiner, W. M. Generic revision of Onychiurinae (Collembola: Onychiuridae) with a cladistic analysis. *Ann. Entomol. Soc. Fr.* **32**, 163–200 (1996).

6. Deharveng, L. Morphologie évolutive des Collemboles Neanurinae en particulier de la lignée Neanurienne. *Trav. Lab. Ecobiol. Arthropodes Edaphiques, Toulouse* **4**(2), 1–63 (1983).

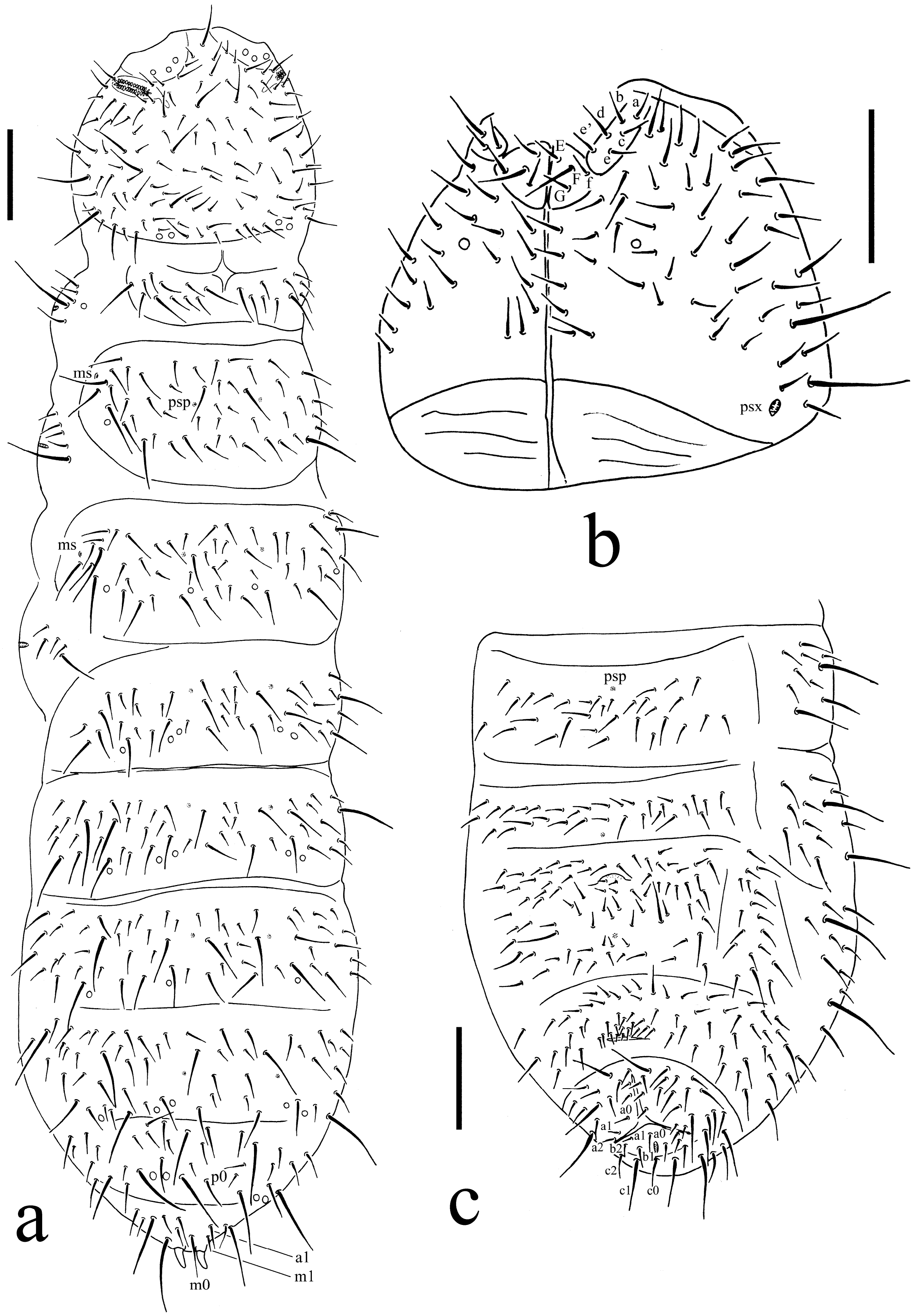

Figure S2. *Protaphorura liui* Sun & Zhang sp. nov.. (a) Dorsal side of body. (b) Ventral side of head. (c) Ventral side of Abd. II–VI. Scales: 0.1 mm.

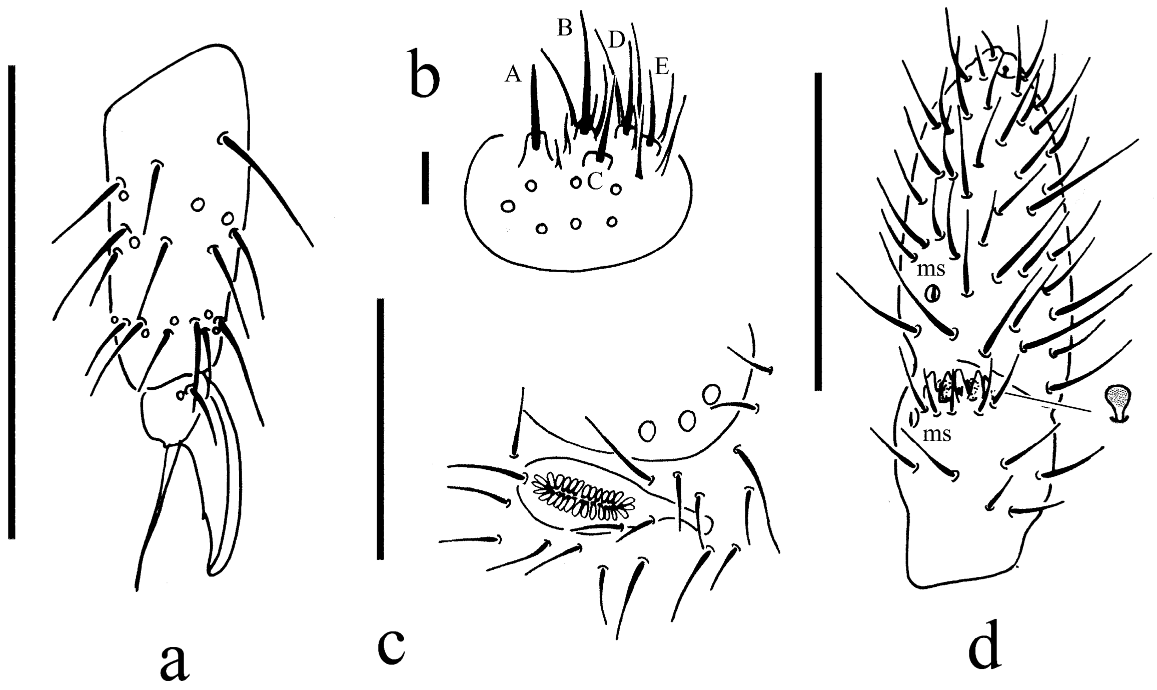

Figure S3. *Protaphorura liui* Sun & Zhang sp. nov.. (a) Distal part of leg. III. (b) Labium. (c) Postantennal organ, showing the sensory club. (d) Ant. III–IV. Scales: 0.1 mm (a, c, d), 0.01 mm (b).

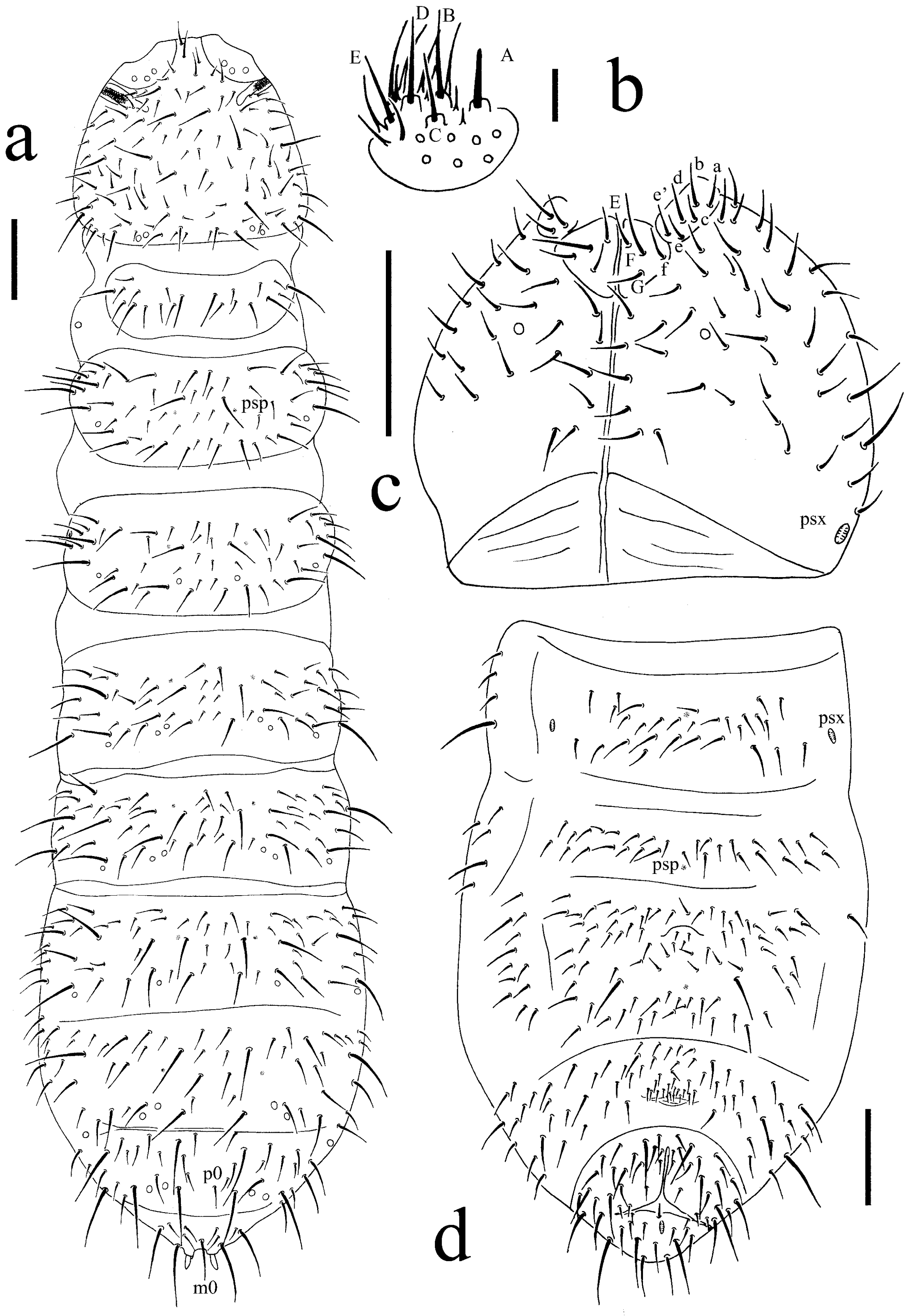

Figure S4. *Protaphorura jiaoheensis* Sun & Zhang sp. nov.. (a) Dorsal side of body. (b) Labium. (c) Ventral side of head. (d) Ventral side of Abd. II–VI. Scales: 0.1 mm (a, c, d), 0.01 mm (b).

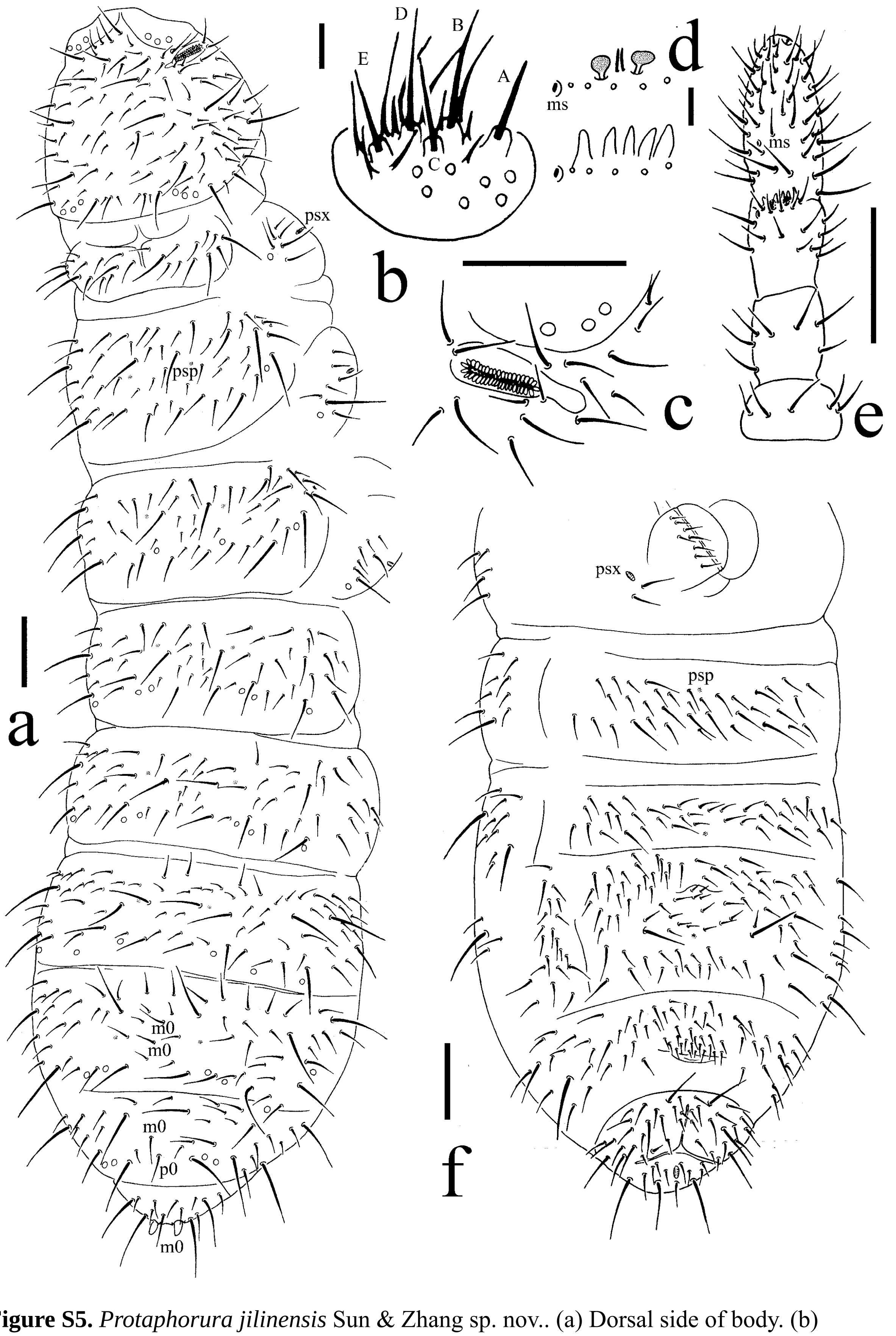

Figure S5. *Protaphorura jilinensis* Sun & Zhang sp. nov.. (a) Dorsal side of body. (b) Labium. (c) Postantennal organ. (d) Sensory organ of Ant. III. (e) Antenna. (f) Ventral side of Abd. I–VI. Scales: 0.1 mm (a, c, e, f), 0.01 mm (b, d).

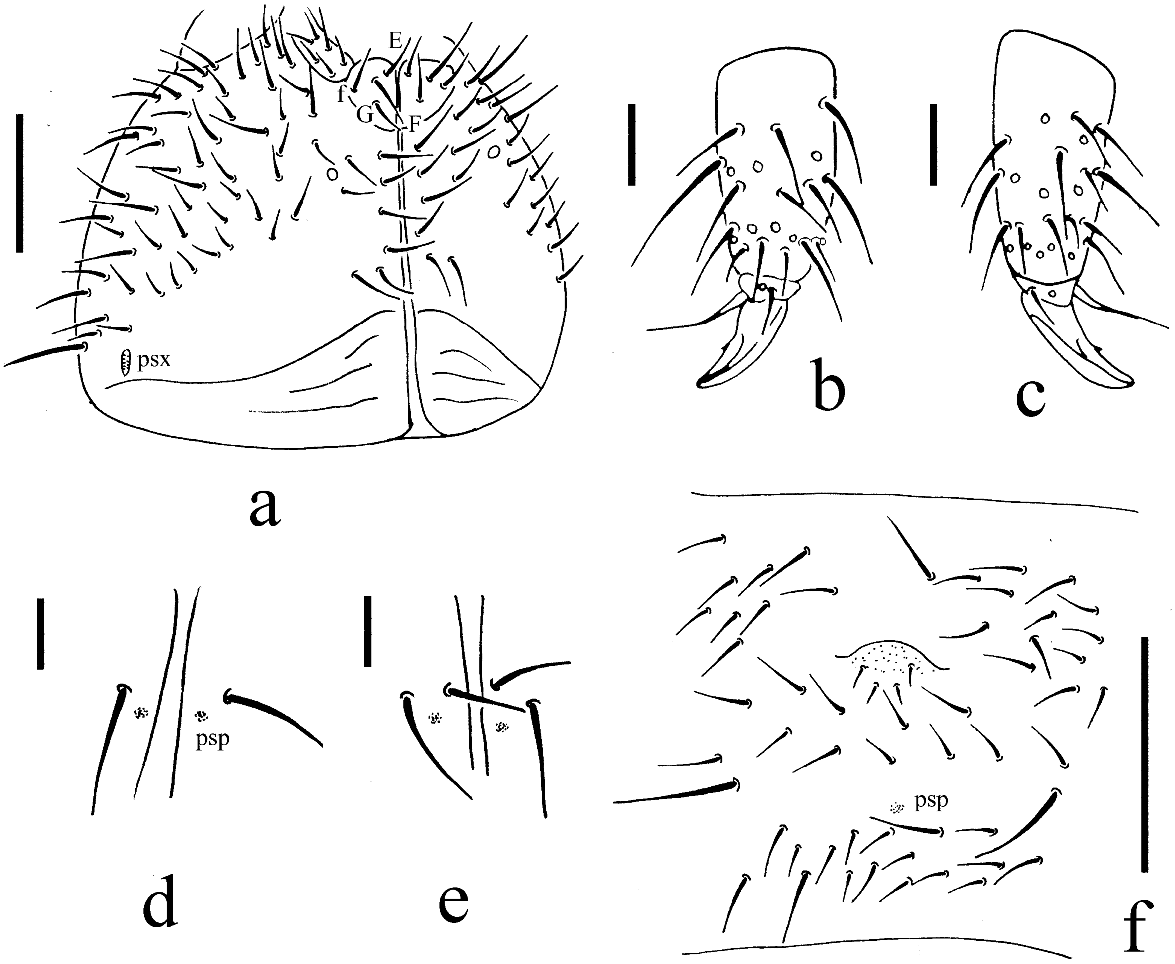

Figure S6. *Protaphorura jilinensis* Sun & Zhang sp. nov.. (a) Ventral side of head. (b) Distal part of leg. I. (c) Distal part of leg. III. (d) Th. sternum II. (e) Th. sternum III. (f) Central part of Abd. sternum IV. Scales: 0.1 mm (a, f), 0.01 mm (b–e).

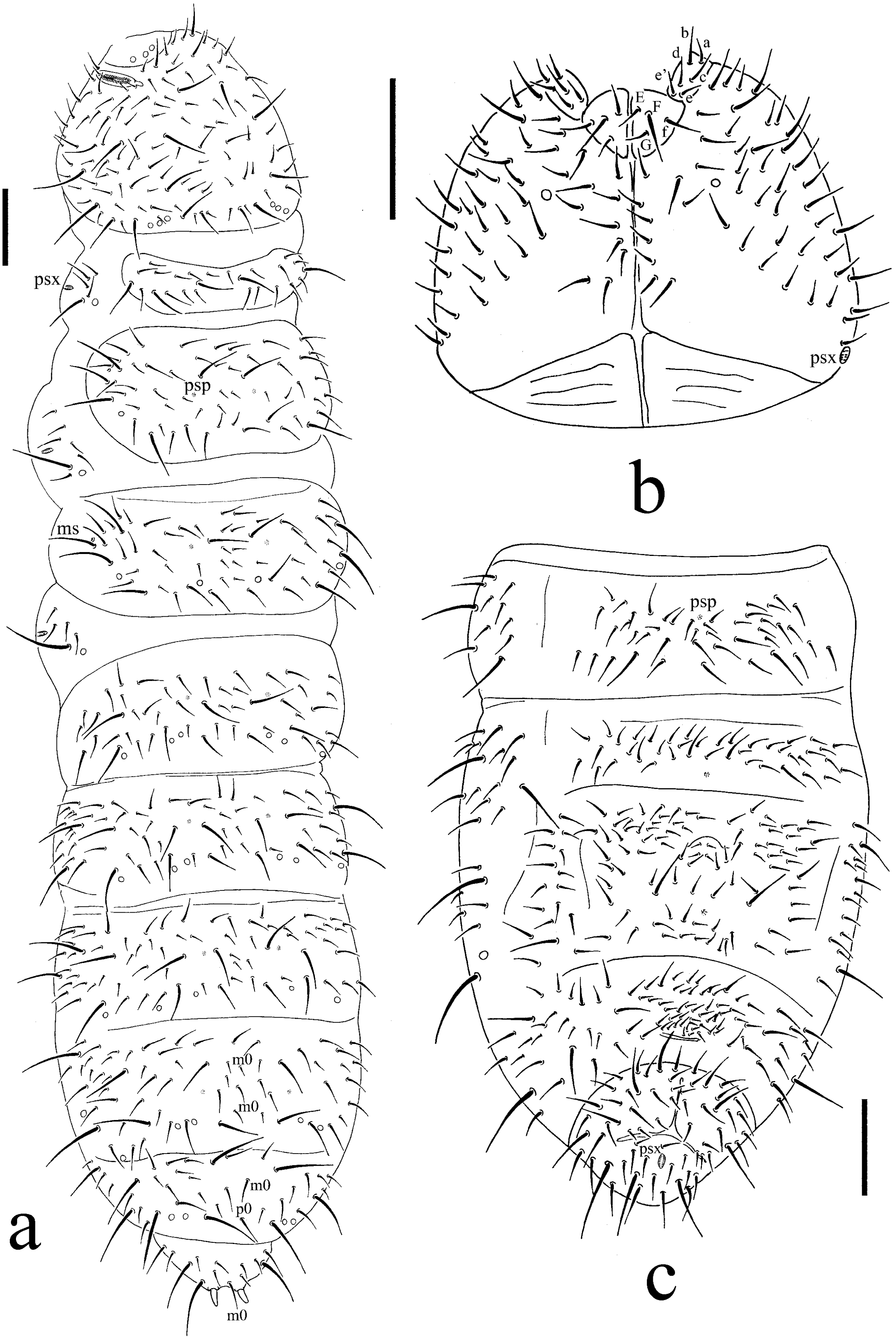

Figure S7. *Protaphorura khanka* Sun & Zhang sp. nov.. (a) Dorsal side of body. (b) Ventral side of head. (c) Ventral side of Abd. II–VI. Scales: 0.1 mm.

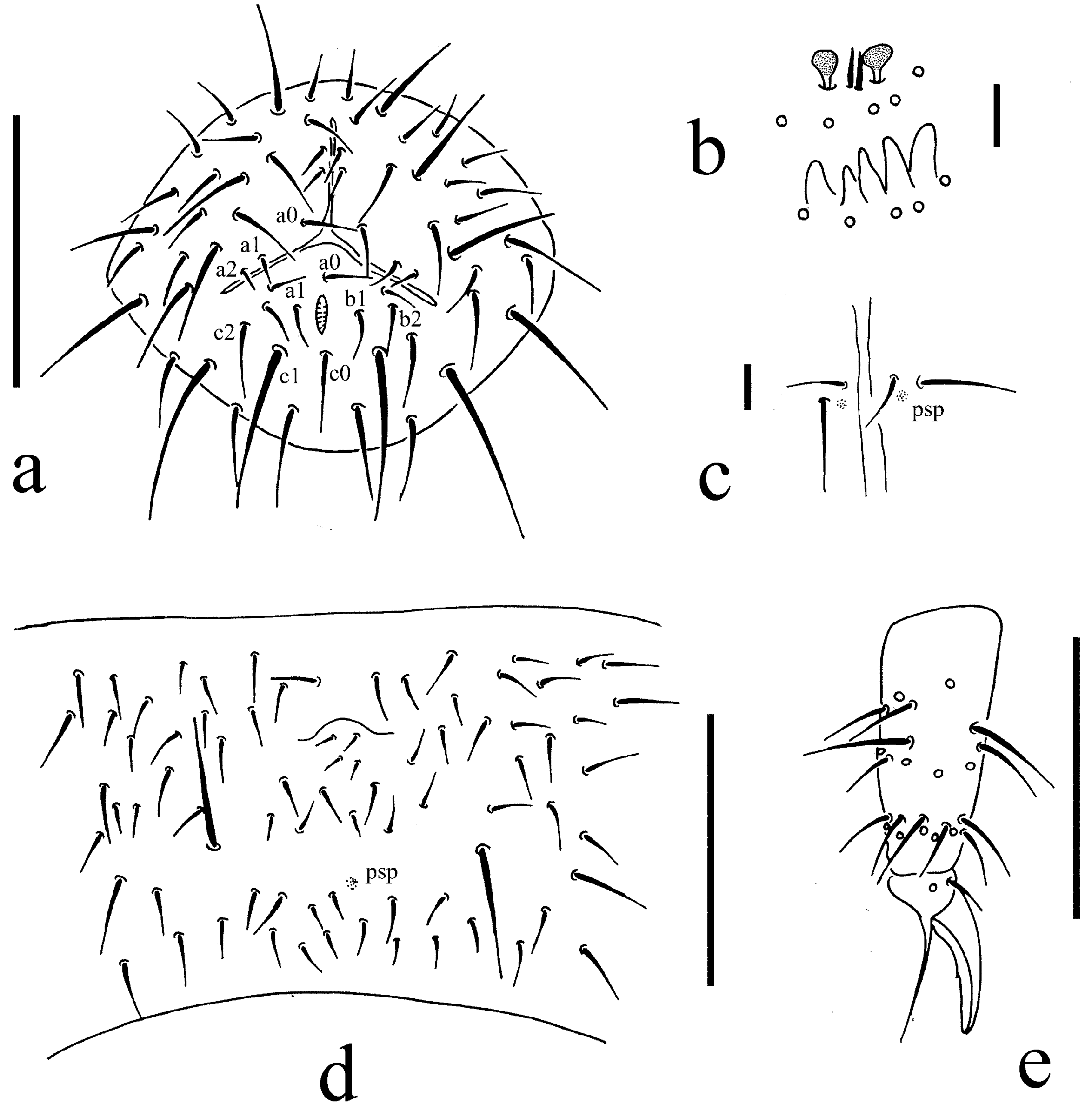

Figure S8. *Protaphorura khanka* Sun & Zhang sp. nov.. (a) Anal valves. (b) Sensory organ of Ant. III. (c) Th. sternum II. (d) Central part of Abd. sternum IV. (e) Distal part of leg. III. Scales: 0.1 mm (a, d, e), 0.01 mm (b–c).

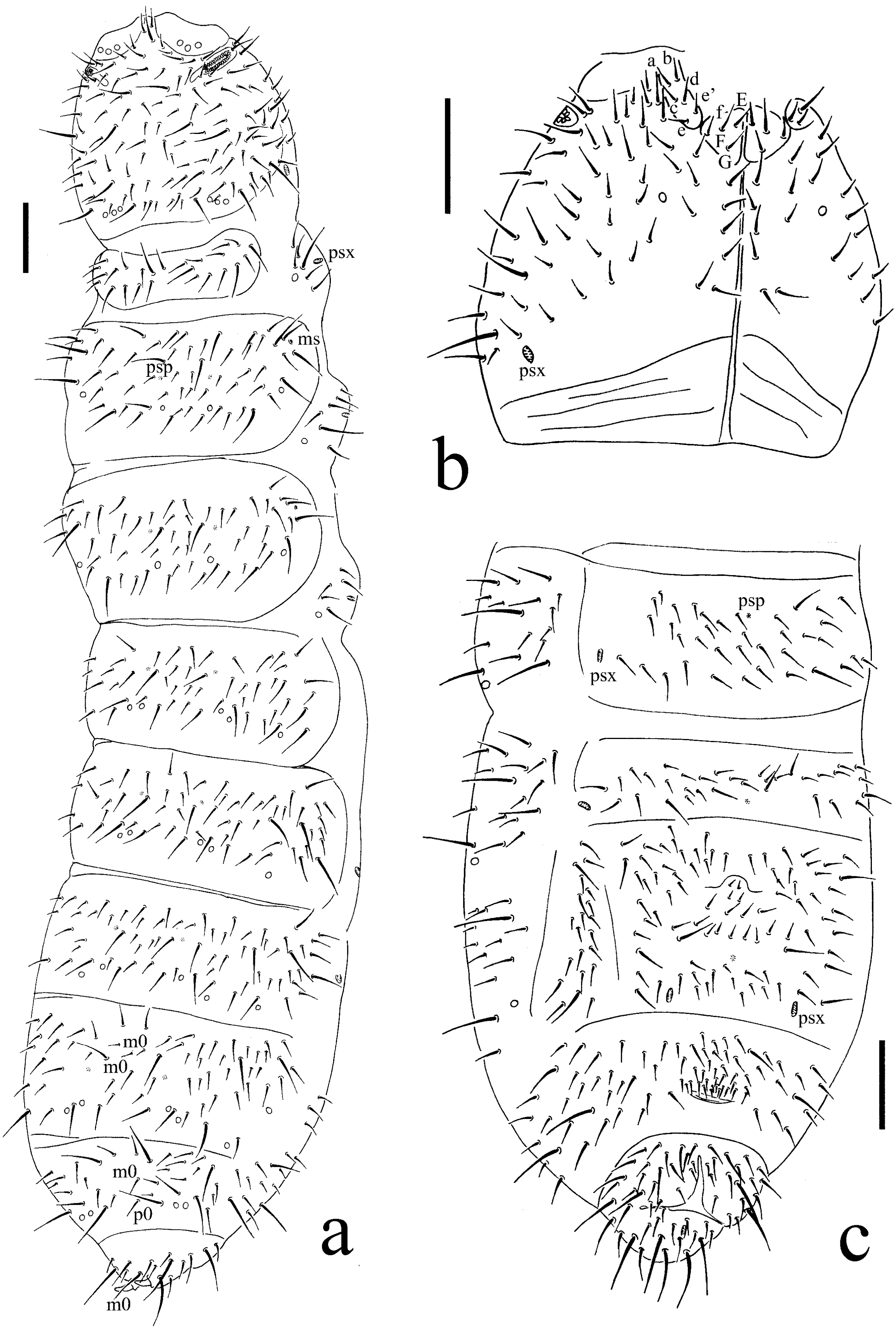

Figure S9. *Protaphorura uniseta* Sun & Zhang sp. nov.. (a) Dorsal side of body. (b) Ventral side of head. (c) Ventral side of Abd. II–VI. Scales: 0.1 mm.

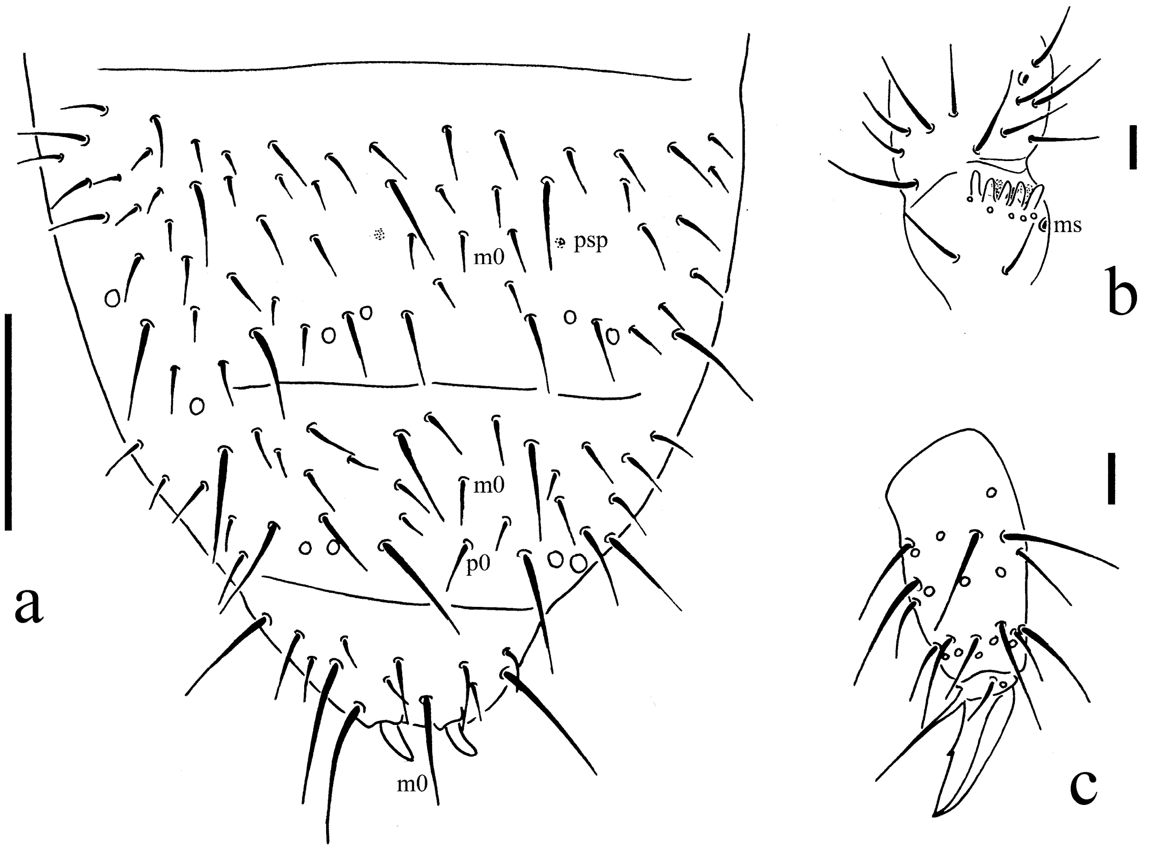

Figure S10. *Protaphorura uniseta* Sun & Zhang sp. nov.. (a) Dorsal side of Abd. IV–VI. (b) Sensory organ of Ant. III. (c) Distal part of leg. III. Scales: 0.1 mm (a), 0.01 mm (b–c).

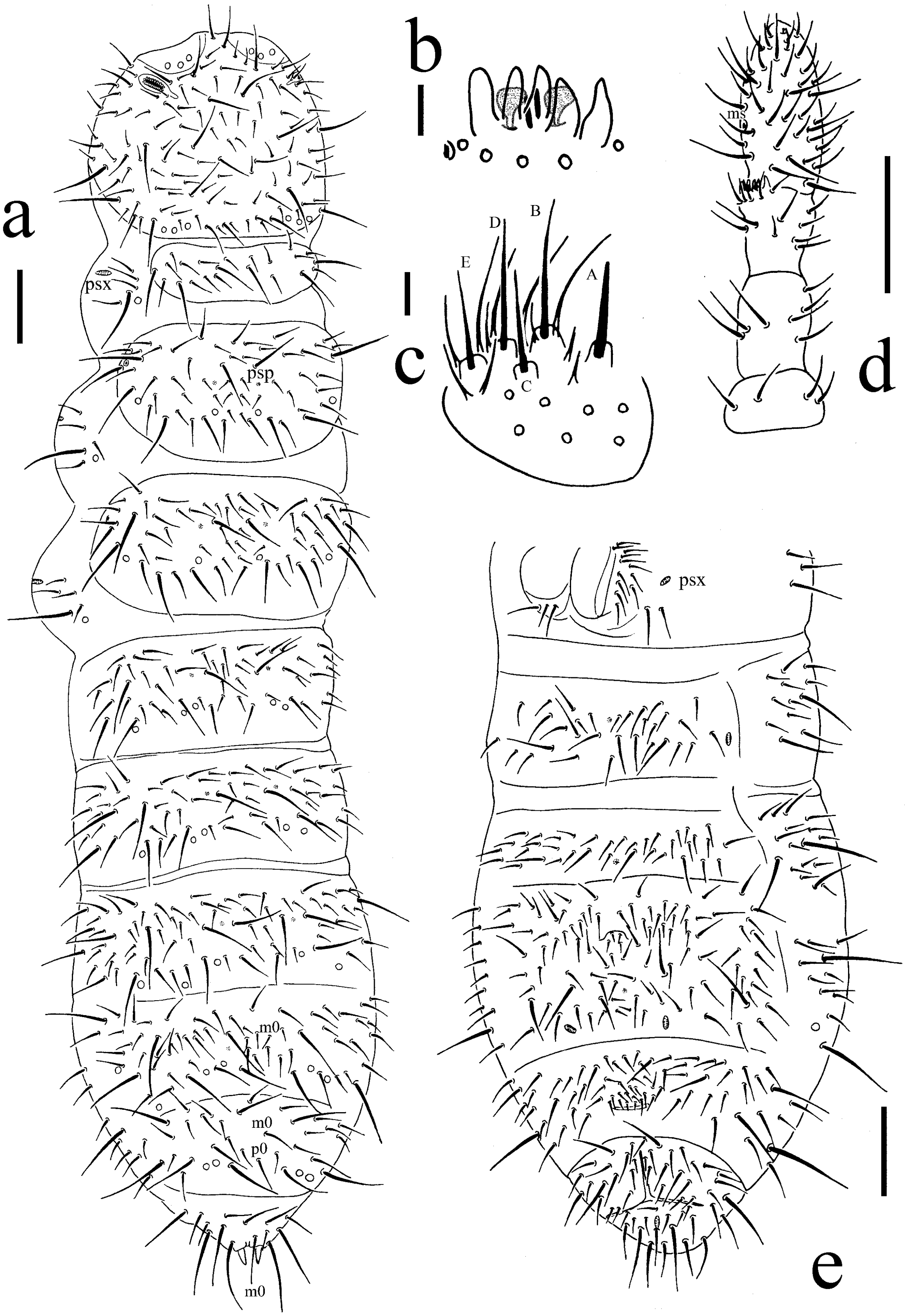

Figure S11. *Protaphorura zhangae* Sun & Zhang sp. nov.. (a) Dorsal side of body. (b) Sensory organ of Ant. III. (c) Labium. (d) Antenna. (e) Ventral side of Abd. I–VI. Scales: 0.1 mm (a, d, e), 0.01 mm (b–c).

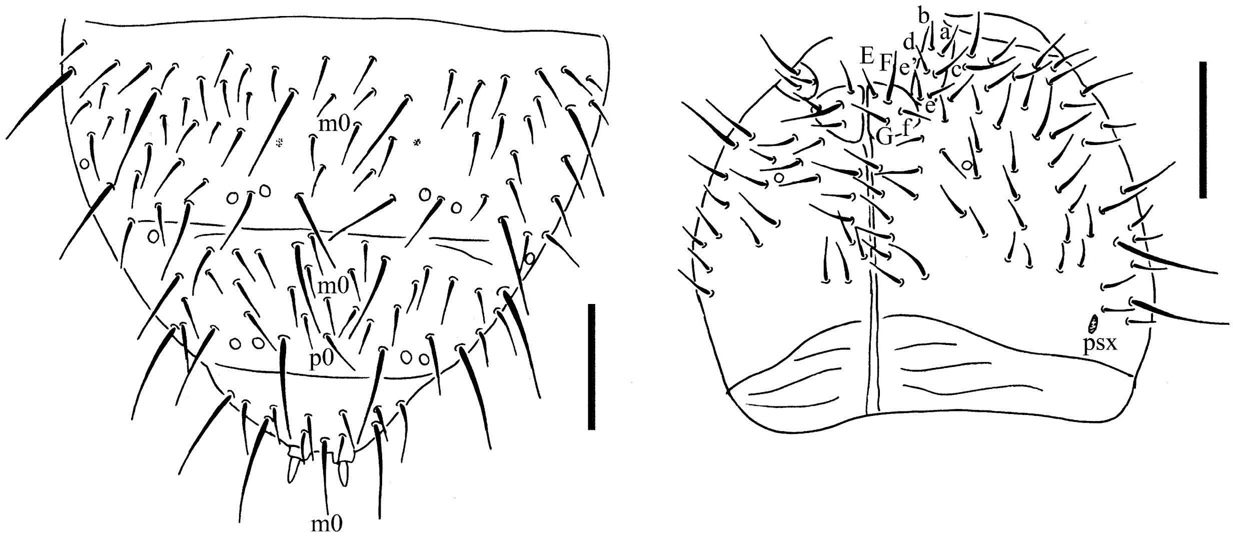

a b

Figure S12. *Protaphorura zhangae* Sun & Zhang sp. nov.. (a) Dorsal side of Abd. IV–VI. (b) Ventral side of head. Scales: 0.1 mm.

Table S1 Intraspecific divergence

Gp 1	0.027637643	
Gp 2	0.058821314	maximum
Gp 3	0.005283276	
Gp 4	0.001960938	
Gp 5	0.000302519	
Gp 6	0	
Gp 7	0.034818682	
Gp 8	0.001523001	
Gp 9	0.009937169	
Gp 10	0.000811462	
Gp 11	n/c	
Gp 12	0.011319116	
Gp 13	0.003823571	
Gp 14	0.000304298	
Gp 15	0.000761202	
Gp 16	0	
Gp 17	0.003814978	
Gp 18	0.023312314	
Gp 19	n/c	
Gp 20	0	
Gp 21	0.001522071	
Gp 22	0.000760746	

Table S2. Interspecific divergence

Gp_1																					
Gp_2	0.14862																				
Gp_3	0.14349	0.17197																			
Gp_4	0.13649	0.13727	0.15123																		
Gp_5	0.13529	0.17667	0.17209	0.15284																	
Gp_6	0.14910	0.15393	0.18276	0.14515	0.18607																
Gp_7	0.15269	0.12215	0.19721	0.14579	0.15785	0.17557															
Gp_8	0.14569	0.16742	0.15910	0.14403	0.15961	0.15688	0.17643														
Gp_9	0.14570	0.13806	0.19560	0.13406	0.17838	0.12417	0.14599	0.18106													
Gp_10	0.14717	0.16172	0.16732	0.11363	0.17745	0.16518	0.16142	0.15634	0.17885												
Gp_11	0.16369	0.17967	0.17429	0.16263	0.16077	0.17237	0.17045	0.12943	0.19966	0.16745											
Gp_12	0.16036	0.17422	0.16258	0.14087	0.18169	0.19188	0.18071	0.14671	0.18532	0.17062	0.15792										
Gp_13	0.15086	0.16877	0.18451	0.12225	0.17059	0.15495	0.16528	0.16589	0.16875	0.12826	0.17190	0.16891									
Gp_14	0.09659	0.16590	0.13632	0.14441	0.12982	0.16420	0.15987	0.16347	0.17760	0.14128	0.16613	0.18373	0.16304								
Gp_15	0.15501	0.17460	0.17080	0.14603	0.17554	0.17410	0.18534	0.15812	0.17441	0.16097	0.16698	0.16606	0.13682	0.15198							
Gp_16	0.10324	0.15946	0.16489	0.14535	0.13365	0.18212	0.15444	0.16897	0.14566	0.16160	0.19138	0.18039	0.16168	0.11456	0.15507						
Gp_17	0.13830	0.15358	0.15458	0.09491	0.14926	0.13458	0.16783	0.14318	0.15283	0.10660	0.15197	0.16836	0.11703	0.13708	0.12626	0.14188					
Gp_18	0.15731	0.17924	0.13722	0.16756	0.17676	0.19607	0.17480	0.16065	0.18449	0.16596	0.16052	0.18664	0.17933	0.16310	0.15360	0.17767	0.15599				
Gp_19	0.13397	0.16351	0.13993	0.14926	0.16457	0.18349	0.16631	0.16583	0.17867	0.15546	0.17049	0.18455	0.18037	0.15325	0.16864	0.15884	0.15111	0.10208			
Gp_20	0.16994	0.15183	0.16830	0.10601	0.18539	0.17026	0.17115	0.15090	0.15206	0.13717	0.18455	0.16959	0.15633	0.17643	0.15639	0.17616	0.13310	0.17312	0.17411		
Gp_21	0.16639	0.16555	0.15478	0.17041	0.19259	0.20081	0.18640	0.16646	0.18252	0.17994	0.17897	0.19617	0.18866	0.16595	0.16624	0.16781	0.16649	0.11147	0.10909	0.18253	
Gp_22	0.14711	0.15356	0.16423	0.08591	0.17153	0.14994	0.16609	0.14188	0.15592	0.13032	0.17699	0.17204	0.15185	0.15962	0.14904	0.16161	0.11610	0.17072	0.16469	0.10179	0.17269

Table S3. Molecular analysis information

ID	Group	Species	Coordinate	GenBank accession number	Voucher specimen	Dorsal pso formula checked after the molecular analysis
HLECYD_5	Gp 7	*P.* cf. *bicampata* (Gisin, 1956)	49.10684° N 119.74769 E	KU508196	+	33/022/33332
HLECYD_6	Gp 7	*P.* cf. *bicampata* (Gisin, 1956)	49.10684° N 119.74769 E	KU508197	+	33/022/33332
HLECYD_1	Gp 7	*P.* cf. *bicampata* (Gisin, 1956)	49.10684° N 119.74769 E	KU508192	–	–
HLECYD_8	Gp 7	*P.* cf. *bicampata* (Gisin, 1956)	49.10684° N 119.74769 E	KU508199	+	33/022/33332
HLECYD_4	Gp 7	*P.* cf. *bicampata* (Gisin, 1956)	49.10684° N 119.74769 E	KU508195	–	–
HLECYD_2	Gp 7	*P.* cf. *bicampata* (Gisin, 1956)	49.10684° N 119.74769 E	KU508193	–	–
HLECYD_3	Gp 7	*P.* cf. *bicampata* (Gisin, 1956)	49.10684° N 119.74769 E	KU508194	+	33/022/33332
HLECYD_7	Gp 7	*P.* cf. *bicampata* (Gisin, 1956)	49.10684° N 119.74769 E	KU508198	+	33/022/33332
EEGNT_1	Gp 7	*P.* cf. *bicampata* (Gisin, 1956)	50.12214° N 120.20458° E	KU508138	+	33/022/33332
EEGNT_2	Gp 7	*P.* cf. *bicampata* (Gisin, 1956)	50.12214° N 120.20458° E	KU508139	+	33/022/33332
EEGNT_3	Gp 7	*P.* cf. *bicampata* (Gisin, 1956)	50.12214° N 120.20458° E	KU508140	+	33/022/33332
EEGNT_4	Gp 7	*P.* cf. *bicampata* (Gisin, 1956)	50.12214° N 120.20458° E	KU508141	+	33/022/33332
EEGNT_5	Gp 7	*P.* cf. *bicampata* (Gisin, 1956)	50.12214° N 120.20458° E	KU508142	+	33/022/33332
EEGNT_8	Gp 7	*P.* cf. *bicampata* (Gisin, 1956)	50.12214° N 120.20458° E	KU508145	+	33/022/33332
AESHGD_1	Gp 2	*P.* cf. *bicampata* (Gisin, 1956)	47.510519° N 120.635001° E	KU508100	–	–
AESHGD_5	Gp 2	*P.* cf. *bicampata* (Gisin, 1956)	47.510519° N 120.635001° E	KU508104	+	33/022/33332+3
SJDQZHBC_3	Gp 2	*P.* cf. *bicampata* (Gisin, 1956)	47.719336° N 133.584393° E	KU508223	+	33/022/33332
SJDQZHBC_4	Gp 2	*P.* cf. *bicampata* (Gisin, 1956)	47.719336° N 133.584393° E	KU508224	–	–
SANJIANGDIQU	Gp 2	*P.* cf. *bicampata* (Gisin, 1956)	47.719336° N 133.584393° E	KU508220	–	–
SJDQZHBC_1	Gp 2	*P.* cf. *bicampata* (Gisin, 1956)	47.719336° N 133.584393° E	KU508221	+	33/022/33332
SJDQZHBC_2	Gp 2	*P.* cf. *bicampata* (Gisin, 1956)	47.719336° N 133.584393° E	KU508222	+	33/022/33332
LD_11_060_2	Gp 6	*P. uniseta* sp. nov.	49.058176° N 127.074391° E	KU508211	–	
LD_11_060_2_2	Gp 6	*P. uniseta* sp. nov.	49.058176° N 127.074391° E	KU508212	+	33/022/33342
EEGNT_7	Gp 6	*P. uniseta* sp. nov.	50.12214° N 120.20458° E	KU508144	+	33/022/33342
EEGNT_6	Gp 6	*P. uniseta* sp. nov.	50.12214° N 120.20458° E	KU508143	+	33/022/33342
CB_12_23_4	Gp 6	*P. uniseta* sp. nov.	42.40093° N 128.10677° E	KU508137	–	–
CB_12_23_2	Gp 6	*P. uniseta* sp. nov.	42.40093° N 128.10677° E	KU508136	–	–
YTLHHJD_9	Gp 9	*P. genheensis* Sun, Chang & Wu, 2015	50.69010° N 121.71912° E	KU508240	+	33/012/33342
YTLHHJD_5	Gp 9	*P. genheensis* Sun, Chang & Wu, 2015	50.69010° N 121.71912° E	KU508236	+	33/012/33342
YTLHHJD_6	Gp 9	*P. genheensis* Sun, Chang & Wu, 2015	50.69010° N 121.71912° E	KU508237	+	33/012/33342
YTLHHJD_10	Gp 9	*P. genheensis* Sun, Chang & Wu, 2015	50.69010° N 121.71912° E	KU508232	+	33/012/33342
GHLYST_10	Gp 9	*P. genheensis* Sun, Chang & Wu, 2015	50.72447° N 121.49123 E	KU508147	–	–
GHLYST_11	Gp 9	*P. genheensis* Sun, Chang & Wu, 2015	50.72447° N 121.49123 E	KU508148	+	33/012/33342
GHLYST_12	Gp 9	*P. genheensis* Sun, Chang & Wu, 2015	50.72447° N 121.49123 E	KU508149	+	33/012/33342
GHLYST_13	Gp 9	*P. genheensis* Sun, Chang & Wu, 2015	50.72447° N 121.49123 E	KU508150	+	33/012/33342

GHLYST_14	Gp 9	*P. genheensis* Sun, Chang & Wu, 2015	50.72447° N 121.49123 E	KU508151	+	33/012/33342
GHLYST_16	Gp 9	*P. genheensis* Sun, Chang & Wu, 2015	50.72447° N 121.49123 E	KU508152	+	33/012/33342
GHLYST_2	Gp 9	*P. genheensis* Sun, Chang & Wu, 2015	50.72447° N 121.49123 E	KU508153	+	33/012/33342
GHLYST_5	Gp 9	*P. genheensis* Sun, Chang & Wu, 2015	50.72447° N 121.49123 E	KU508156	+	33/012/33342
GHLYST_7	Gp 9	*P. genheensis* Sun, Chang & Wu, 2015	50.72447° N 121.49123 E	KU508157	+	33/012/33342
HHEJD_1	Gp 15	*P. zhangae* sp. nov.	48.13709° N 119.76073° E	KU508184	+	33/022/33342
HHEJD_2	Gp 15	*P. zhangae* sp. nov.	48.13709° N 119.76073° E	KU508185	+	33/022/33342
HHEJD_3	Gp 15	*P. zhangae* sp. nov.	48.13709° N 119.76073° E	KU508186	+	33/022/33342
HHEJD_4	Gp 15	*P. zhangae* sp. nov.	48.13709° N 119.76073° E	KU508187	+	33/022/33342
HHEJD_5	Gp 15	*P. zhangae* sp. nov.	48.13709° N 119.76073° E	KU508188	+	33/022/33342
HHEJD_6	Gp 15	*P. zhangae* sp. nov.	48.13709° N 119.76073° E	KU508189	+	33/022/33342
HHEJD_7	Gp 15	*P. zhangae* sp. nov.	48.13709° N 119.76073° E	KU508190	+	33/022/33342
HHEJD_8	Gp 15	*P. zhangae* sp. nov.	48.13709° N 119.76073° E	KU508191	+	33/022/33342
GMX_MES_4	Gp 13	?	45.730317° N 126.647861° E	KU508170	+	33/022/33342
GMX_MES_5	Gp 13	?	45.730317° N 126.647861° E	KU508171	+	33/022/33342
GMX_MES_6	Gp 13	?	45.730317° N 126.647861° E	KU508172	+	33/022/33342
GMX_MES_7	Gp 13	?	45.730317° N 126.647861° E	KU508173	+	33/022/33342
GMX_3	Gp 10	*P.* cf. *taimyrica* (Martynova, 1976)	47.14932° N 128.872301° E	KU508160	+	33/01+22/33342
GMX_4	Gp 10	*P.* cf. *taimyrica* (Martynova, 1976)	47.14932° N 128.872301° E	KU508161	+	33/022/33342
GMX_5	Gp 10	*P.* cf. *taimyrica* (Martynova, 1976)	47.14932° N 128.872301° E	KU508162	+	33/022/33342
GMX_6	Gp 10	*P.* cf. *taimyrica* (Martynova, 1976)	47.14932° N 128.872301° E	KU508163	+	33/022/33342
GMX_9	Gp 10	*P.* cf. *taimyrica* (Martynova, 1976)	47.14932° N 128.872301° E	KU508166	+	33/022/33342
GMX_8	Gp 10	*P.* cf. *taimyrica* (Martynova, 1976)	47.14932° N 128.872301° E	KU508165	+	33/01+22/33342
JHKYLD_2	Gp 17	?	43.80567° N 127.17435° E	KU508202	–	–
JHKYLD_3	Gp 17	?	43.80567° N 127.17435° E	KU508203	–	–
JHKYLD_4	Gp 17	?	43.80567° N 127.17435° E	KU508204	+	33+2/01+22/33342
JHKYLD_9	Gp 17	?	43.80567° N 127.17435° E	KU508209	+	33/022/33342
C0018_1	Gp 4	*P. jilinensis* sp. nov.	41.76° N 127.94° E	KU508109	+	33/012/33342
C0018_2	Gp 4	*P. jilinensis* sp. nov.	41.76° N 127.94° E	KU508110	+	33/012/33342
C0018_3	Gp 4	*P. jilinensis* sp. nov.	41.76° N 127.94° E	KU508111	+	33/012/33342
C0018_4	Gp 4	*P. jilinensis* sp. nov.	41.76° N 127.94° E	KU508112	+	33/012/33342
C0018_5	Gp 4	*P. jilinensis* sp. nov.	41.76° N 127.94° E	KU508113	–	–
C0018_6	Gp 4	*P. jilinensis* sp. nov.	41.76° N 127.94° E	KU508114	+	33/012/33342
C0018_7	Gp 4	*P. jilinensis* sp. nov.	41.76° N 127.94° E	KU508115	+	33/012/33342
C0018_8	Gp 4	*P. jilinensis* sp. nov.	41.76° N 127.94° E	KU508116	+	33/012/33342
YTLHHJD_2	Gp 22	*P.* cf. *taimyrica* (Martynova, 1976)	50.69010° N 121.71912° E	KU508233	+	33/022/33342
YTLHHJD_3	Gp 22	*P.* cf. *taimyrica* (Martynova, 1976)	50.69010° N 121.71912° E	KU508234	–	–
YTLHHJD_7	Gp 22	*P.* cf. *taimyrica* (Martynova, 1976)	50.69010° N 121.71912° E	KU508238	+	33/022/33342
YTLHHJD_8	Gp 22	*P.* cf. *taimyrica* (Martynova, 1976)	50.69010° N 121.71912° E	KU508239	+	33/022/33342
XINKAILINDI	Gp 20	*P. khanka* sp. nov.	45.373181° N 132.332138° E	KU508225	–	–

XKLD_1	Gp 20	*P. khanka* sp. nov.	45.373181° N 132.332138° E	KU508226	+	33/012/33342
XKLD_2	Gp 20	*P. khanka* sp. nov.	45.373181° N 132.332138° E	KU508227	+	33/012/33342
XKLD_3	Gp 20	*P. khanka* sp. nov.	45.373181° N 132.332138° E	KU508228	+	33/012/33342
XKLD_6	Gp 20	*P. khanka* sp. nov.	45.373181° N 132.332138° E	KU508230	+	33/012/33342
XKLD_5	Gp 20	*P. khanka* sp. nov.	45.373181° N 132.332138° E	KU508229	+	33/012/33342
HCGSHD_1	Gp 14	*P. liui* sp. nov.	43.07765° N 128.01729° E	KU508174	–	–
HCGSHD_2	Gp 14	*P. liui* sp. nov.	43.07765° N 128.01729° E	KU508176	–	–
HCGSHD_3	Gp 14	*P. liui* sp. nov.	43.07765° N 128.01729° E	KU508177	+	32/012/33232
HCGSHD_4	Gp 14	*P. liui* sp. nov.	43.07765° N 128.01729° E	KU508178	+	32/012/33232
HCGSHD_5	Gp 14	*P. liui* sp. nov.	43.07765° N 128.01729° E	KU508179	+	32/012/33232
HCGSHD_6	Gp 14	*P. liui* sp. nov.	43.07765° N 128.01729° E	KU508180	+	32/012/33232
HCGSHD_7	Gp 14	*P. liui* sp. nov.	43.07765° N 128.01729° E	KU508181	–	–
HCGSHD_8	Gp 14	*P. liui* sp. nov.	43.07765° N 128.01729° E	KU508182	+	32/012/33232
HCGSHD_9	Gp 14	*P. liui* sp. nov.	43.07765° N 128.01729° E	KU508183	+	32/012/33232
HCGSHD_10	Gp 14	*P. liui* sp. nov.	43.07765° N 128.01729° E	KU508175	+	32/012/33232
14SY_Protaphorura_4	Gp 1	*P.* cf. *changbaiensis* Sun, Zhang & Wu, 2013	41.85547° N 123.42595° E	KU508099	+	32/012/33232
14SY_5_Protaphorura	Gp 1	*P.* cf. *changbaiensis* Sun, Zhang & Wu, 2013	41.85547° N 123.42595° E	KU508097	+	32/012/33232
14SY_6_Protaphorura	Gp 1	*P.* cf. *changbaiensis* Sun, Zhang & Wu, 2013	41.85547° N 123.42595° E	KU508098	+	32/012/33232
JHKYLD_1	Gp 16	*P. jiaoheensis* sp. nov.	43.80567° N 127.17435° E	KU508200	–	–
JHKYLD_5	Gp 16	*P. jiaoheensis* sp. nov.	43.80567° N 127.17435° E	KU508205	+	32/012/33232
JHKYLD_6	Gp 16	*P. jiaoheensis* sp. nov.	43.80567° N 127.17435° E	KU508206	+	32/012/33232
JHKYLD_7	Gp 16	*P. jiaoheensis* sp. nov.	43.80567° N 127.17435° E	KU508207	+	32/012/33232
JHKYLD_8	Gp 16	*P. jiaoheensis* sp. nov.	43.80567° N 127.17435° E	KU508208	+	32/012/33232
JHKYLD_10	Gp 16	*P. jiaoheensis* sp. nov.	43.80567° N 127.17435° E	KU508201	+	32/012/33232
CB_12_16_2	Gp 5	*P. changbaiensis* Sun, Zhang & Wu, 2013	42.270789° N 128.146863° E	KU508129	–	–
CB_12_16_3	Gp 5	*P. changbaiensis* Sun, Zhang & Wu, 2013	42.270789° N 128.146863° E	KU508130	+	32/012/33232
CB_12_08_6	Gp 5	*P. changbaiensis* Sun, Zhang & Wu, 2013	42.270789° N 128.146863° E	KU508121	–	–
CB_12_09_1	Gp 5	*P. changbaiensis* Sun, Zhang & Wu, 2013	42.270789° N 128.146863° E	KU508122	–	–
CB_12_08_5	Gp 5	*P. changbaiensis* Sun, Zhang & Wu, 2013	42.270789° N 128.146863° E	KU508120	–	–
CB_12_08_4	Gp 5	*P. changbaiensis* Sun, Zhang & Wu, 2013	42.270789° N 128.146863° E	KU508119	–	–
CB_12_08_1	Gp 5	*P. changbaiensis* Sun, Zhang & Wu, 2013	42.270789° N 128.146863° E	KU508117	–	–
CB_12_08_3	Gp 5	*P. changbaiensis* Sun, Zhang & Wu, 2013	42.270789° N 128.146863° E	KU508118	–	–
CB_12_09_3	Gp 5	*P. changbaiensis* Sun, Zhang & Wu, 2013	42.270789° N 128.146863° E	KU508123	–	–
CB_12_09_4	Gp 5	*P. changbaiensis* Sun, Zhang & Wu, 2013	42.270789° N 128.146863° E	KU508124	–	–
CB_12_09_5	Gp 5	*P. changbaiensis* Sun, Zhang & Wu, 2013	42.270789° N 128.146863° E	KU508125	–	–
CB_12_09_6	Gp 5	*P. changbaiensis* Sun, Zhang & Wu, 2013	42.270789° N 128.146863° E	KU508126	–	–
CB_12_16_1	Gp 5	*P. changbaiensis* Sun, Zhang & Wu, 2013	42.270789° N 128.146863° E	KU508127	+	32/012/33232

CB_12_16_1_2	Gp 5	*P. changbaiensis* Sun, Zhang & Wu, 2013	42.270789° N 128.146863° E	KU508128	–	–
CB_12_16_3_2	Gp 5	*P. changbaiensis* Sun, Zhang & Wu, 2013	42.270789° N 128.146863° E	KU508131	–	–
CB_12_16_4	Gp 5	*P. changbaiensis* Sun, Zhang & Wu, 2013	42.270789° N 128.146863° E	KU508132	–	–
CB_12_16_4_2	Gp 5	*P. changbaiensis* Sun, Zhang & Wu, 2013	42.270789° N 128.146863° E	KU508133	+	32/012/33232
CB_12_16_5	Gp 5	*P. changbaiensis* Sun, Zhang & Wu, 2013	42.270789° N 128.146863° E	KU508134	+	32/012/33232
CB_12_16_6	Gp 5	*P. changbaiensis* Sun, Zhang & Wu, 2013	42.270789° N 128.146863° E	KU508135	+	32/012/33232
GMX_MES_1	Gp 12	*P.maoerensis* Sun, Wu & Gao, 2013	45.730317° N 126.647861° E	KU508167	+	43/022/33353
GMX_MES_2	Gp 12	*P.maoerensis* Sun, Wu & Gao, 2013	45.730317° N 126.647861° E	KU508168	+	43/022/33353
GMX_MES_3	Gp 12	*P.maoerensis* Sun, Wu & Gao, 2013	45.730317° N 126.647861° E	KU508169	+	43/022/33353
GMX_7	Gp 11	?	47.14932° N 128.872301° E	KU508164	+	33/022/33342
GHLYST_1	Gp 8	*P.* cf. *kaszabi* (Dunger, 1978)	50.72447° N 121.49123 E	KU508146	+	33/033/33342
GHLYST_8	Gp 8	*P.* cf. *kaszabi* (Dunger, 1978)	50.72447° N 121.49123 E	KU508158	+	33/033/33342
GHLYST_3	Gp 8	*P.* cf. *kaszabi* (Dunger, 1978)	50.72447° N 121.49123 E	KU508154	–	–
GHLYST_4	Gp 8	*P.* cf. *kaszabi* (Dunger, 1978)	50.72447° N 121.49123 E	KU508155	+	33/033/33342
GHLYST_9	Gp 8	*P.* cf. *kaszabi* (Dunger, 1978)	50.72447° N 121.49123 E	KU508159	+	33/033/33342
AESHGD_2	Gp 3	*P.* cf. *subarctica* Martynova, 1976	47.510519° N 120.635001° E	KU508102	–	–
AESHGD_3	Gp 3	*P.* cf. *subarctica* Martynova, 1976	47.510519° N 120.635001° E	KU508103	–	–
AESHGD_6	Gp 3	*P.* cf. *subarctica* Martynova, 1976	47.510519° N 120.635001° E	KU508105	+	32/022/33332
AESHGD_7	Gp 3	*P.* cf. *subarctica* Martynova, 1976	47.510519° N 120.635001° E	KU508106	+	32/022/33332
AESHGD_8	Gp 3	*P.* cf. *subarctica* Martynova, 1976	47.510519° N 120.635001° E	KU508107	+	32/022/33332
AESHGD_9	Gp 3	*P.* cf. *subarctica* Martynova, 1976	47.510519° N 120.635001° E	KU508108	+	32/022/33332
AESHGD_10	Gp 3	*P.* cf. *subarctica* Martynova, 1976	47.510519° N 120.635001° E	KU508101	+	32/022/33332
LD_11_061_1	Gp 19	?	49.058176° N 127.074391° E	KU508214	+	32/022/33342
YTLHHJD_1	Gp 21	?	50.69010° N 121.71912° E	KU508231	+	32/022/33342
YTLHHJD_4	Gp 21	?	50.69010° N 121.71912° E	KU508235	+	32/022/33342
LD_11_061_4	Gp 18	?	49.058176° N 127.074391° E	KU508217	+	33/022/33343
LD_11_061_5	Gp 18	?	49.058176° N 127.074391° E	KU508218	+	33/022/33343
LD_11_060_1_2	Gp 18	?	49.058176° N 127.074391° E	KU508210	+	33+2/022/33343
LD_11_060_3_2	Gp 18	?	49.058176° N 127.074391° E	KU508213	+	33/022/33343
LD_11_061_2	Gp 18	?	49.058176° N 127.074391° E	KU508215	+	33/022/33343
LD_11_061_3	Gp 18	?	49.058176° N 127.074391° E	KU508216	+	33/022/33343
LD_11_061_6	Gp 18	?	49.058176° N 127.074391° E	KU508219	+	33/022/33343

Note: "–" means that the skin of the specimen is missing after the molecular work; "vouchers" are the specimens which have been examined for pso formula; the character status of the deformities individuals are labled in red.

Table S4. Geographical distances between populations

	41.76° N 127.94° E	41.85547° N 123.42595° E	42.270789° N 128.146863° E	42.40093° N 128.10677° E	43.07765° N 128.01729° E	43.80567° N 127.17435° E	45.373181° N 132.332138° E	45.730317° N 126.647861° E	47.14932° N 128.872301° E	47.510519° N 120.635001° E	47.719336° N 133.584393° E	48.13709° N 119.76073° E	49.058176° N 127.074391° E	49.10684° N 119.74769° E	50.12214° N 120.20458° E	50.69010° N 121.71912° E	50.72447° N 121.49123° E
41.76° N 127.94° E	0	375.2228	59.2661	72.5163	146.5046	235.7253	535.6053	453.225	603.4338	861.9606	798.5158	957.5101	813.9275	1036.8216	1104.8637	1101.6065	1112.6491
41.85547° N 123.42595° E	375.2228	0	393.4059	391.6909	401.2019	375.2624	817.6359	502.4866	730.1119	666.1159	1033.3621	755.3163	849.5913	855.4244	951.8994	990.7264	996.9633
42.270789° N 128.146863° E	59.2661	393.4059	0	14.8289	90.2583	188.0284	481.7154	402.7381	545.1643	830.6439	741.5229	925.8668	758.9756	1001.4223	1065.1972	1057.115	1068.5999
42.40093° N 128.10677° E	72.5163	391.6909	14.8289	0	75.5315	173.5357	473.5288	387.9466	531.1362	817.8451	731.3504	912.9922	744.2569	987.9941	1051.2229	1042.6513	1054.1741
43.07765° N 128.01729° E	146.5046	401.2019	90.2583	75.5315	0	105.8235	428.7606	314.2897	457.4672	759.6946	675.004	854.153	668.7266	925.278	984.5363	972.1035	983.9446
43.80567° N 127.17435° E	235.7253	375.2624	188.0284	173.5357	105.8235	0	444.9622	217.9024	394.5929	654.8299	661.3211	749.0161	583.9175	819.4683	879.2105	869.116	880.6563
45.373181° N 132.332138° E	535.6053	817.6359	481.7154	473.5288	428.7606	444.9622	0	445.5408	331.8074	928.7167	277.9157	1007.0715	571.0959	1037.6714	1049.746	986.3541	1001.988
45.730317° N 126.647861° E	453.225	502.4866	402.7381	387.9466	314.2897	217.9024	445.5408	0	232.5802	501.0828	574.2535	588.4599	371.3832	641.4851	685.31	661.7337	674.2903
47.14932° N 128.872301° E	603.4338	730.1119	545.1643	531.1362	457.4672	394.5929	331.8074	232.5802	0	623.6286	361.0133	692.954	250.9398	712.7343	718.6963	655.1743	670.675
47.510519° N 120.635001° E	861.9606	666.1159	830.6439	817.8451	759.6946	654.8299	928.7167	501.0828	623.6286	0	972.6642	95.597	507.7395	189.3117	292.142	362.3491	362.8467
47.719336° N 133.584393°	798.5158	1033.3621	741.5229	731.3504	675.004	661.3211	277.9157	574.2535	361.0133	972.6642	0	1032.6769	504.4225	1034.3544	1014.766	924.3283	940.8532

E																	
48.13709° N 119.76073° E	957.5101	755.3163	925.8668	912.9922	854.153	749.0161	1007.0715	588.4599	692.954	95.597	1032.6769	0	548.8587	107.8428	223.1246	317.4879	313.9279
49.058176° N 127.074391° E	813.9275	849.5913	758.9756	744.2569	668.7266	583.9175	571.0959	371.3832	250.9398	507.7395	504.4225	548.8587	0	535.0407	510.4084	425.4637	441.7801
49.10684° N 119.74769° E	1036.8216	855.4244	1001.4223	987.9941	925.278	819.4683	1037.6714	641.4851	712.7343	189.3117	1034.3544	107.8428	535.0407	0	117.6509	225.9777	219.1975
50.12214° N 120.20458° E	1104.8637	951.8994	1065.1972	1051.2229	984.5363	879.2105	1049.746	685.31	718.6963	292.142	1014.766	223.1246	510.4084	117.6509	0	124.8323	113.353
50.69010° N 121.71912° E	1101.6065	990.7264	1057.115	1042.6513	972.1035	869.116	986.3541	661.7337	655.1743	362.3491	924.3283	317.4879	425.4637	225.9777	124.8323	0	16.5456
50.72447° N 121.49123° E	1112.6491	996.9633	1068.5999	1054.1741	983.9446	880.6563	1001.988	674.2903	670.675	362.8467	940.8532	313.9279	441.7801	219.1975	113.353	16.5456	0

Note: the unit of the number is kilometer (km).

Table S5. The number of putative species delimited using different distance thresholds.

Morphospecies Group	Dorsal pso	Number of pso on subcoxae 1 of legs I-III	Locality	10 km	20 km	50 km	100 km	200 km	300 km	400 km	500 km
MG1	32/012/33232	1,0,0	41.85547° N 123.42595° E; 42.270789° N 128.146863° E; 43.07765° N 128.01729° E; 43.80567° N 127.17435° E	4	4	4	3	2	2	2	1
MG2	32/022/33332	1,1,1	47.510519° N 120.635001° E	1	1	1	1	1	1	1	1
MG3	32/022/33342	1,1,1	49.058176° N 127.074391° E; 50.69010° N 121.71912° E	2	2	2	2	2	2	2	1
MG4	33/012/33342	1,1,1	41.76° N 127.94° E; 50.69010° N 121.71912° E; 50.72447° N 121.49123 E; 45.373181° N 132.332138° E	4	3	3	3	3	3	3	3
MG5	33/022/33332	0,0,0	47.719336° N 133.584393° E; 47.510519° N 120.635001° E; 49.10684° N 119.74769 E; 50.12214° N 120.20458° E	4	4	4	4	2	2	2	2

MG6	33/022/33342	1,1,1	42.40093° N 128.10677° E;<br>49.058176° N 127.074391° E;<br>50.12214° N 120.20458° E;<br>47.14932° N 128.872301° E;<br>45.730317° N 126.647861° E;<br>48.13709° N 119.76073° E;<br>43.80567° N 127.17435° E;<br>50.69010° N 121.71912° E	8	8	8	8	6	2	2	1
MG7	33/022/33342	1,0,0	47.14932° N 128.872301° E	1	1	1	1	1	1	1	1
MG8	33/022/33343	1,1,1	49.058176° N 127.074391° E	1	1	1	1	1	1	1	1
MG9	33/033/33342	1,1,1	50.72447° N 121.49123° E	1	1	1	1	1	1	1	1
MG10	43/022/33353	1,0,0	45.730317° N 126.647861° E	1	1	1	1	1	1	1	1
Total number of putative species				27	26	26	25	20	16	16	13

Note: the numbers in the table means that the number of putative species delimited using the distance thresholds 10, 20, 50, 100, 200, 300, 400, 500 kilometers respectively.

Table S6. The main diagnostic characters of *Protaphoura* from northeast China.

Group	Species	Dorsal pso	Ventral psx	A	B	C	D
Gp 1	*P.* cf. *changbaiensis* Sun, Zhang & Wu, 2013	32/012/33232	0/000/100001^{m}	1,0,0	1+1	absent	parallel
Gp 2	*P.* cf. *bicampata* (Gisin, 1956)	33/022/33332	1/000/111100	0,0,0	2+2	present	convergent
Gp 3	P. cf. *subarctica* Martynova, 1976	32/022/33332	0/000/111101^{m}	1,1,1	2+2	absent	convergent
Gp 4	*P. jilinensis* sp. nov.	33/012/33342	1/000/100001^{m}	1,1,1	1+1	absent	parallel
Gp 5	*P. changbaiensis* Sun, Zhang & Wu, 2013	32/012/33232	0/000/100001^{m}	1,0,0	1+1	absent	parallel
Gp 6	*P. uniseta* sp. nov.	33/022/33342	1/000/111101^{m}	1,1,1	1+1	absent	parallel
Gp 7	*P.* cf. *bicampata* (Gisin, 1956)	33/022/33332	1/000/110100	0,0,0	2+2	present	convergent
Gp 8	*P.* cf. *kaszabi* (Dunger, 1978)	33/033/33342	1/000/110001^{m}	1,1,1	1+1	absent	parallel
Gp 9	*P. genheensis* Sun, Chang & Wu, 2015	33/012/33342	1/000/111101^{m}	1,1,1	1+1	absent	parallel
Gp 10	*P.* cf. *taimyrica* (Martynova, 1976)	33/022/33342	1/000/110001^{m}	1,1,1	1+1	absent	parallel
Gp 11	?	33/022/33342	*	1,0,0	1+1	absent	parallel

Gp 12	*P. maoerensis* Sun, Wu & Gao, 2013	43/022/33353	1/000/110000	1,0,0	2+2	absent	parallel
Gp 13	?	33/022/33342	1/000/100001^{m}	1,1,1	1+1	absent	parallel
Gp 14	*P. liui* sp. nov.	32/012/33232	1/000/100001^{m}	1,0,0	1+1	absent	parallel
Gp 15	*P. zhangae* sp. nov.	33/022/33342	1/000/110101^{m}	1,1,1	1+1	absent	parallel
Gp 16	*P. jiaoheensis* sp. nov.	32/012/33232	1/000/110001^{m}	1,0,0	1+1	absent	parallel
Gp 17	?	33/022/33342	1/000/100001^{m}	1,1,1	1+1	absent	parallel
Gp 18	?	33/022/33343	*	1,1,1	*	absent	parallel
Gp 19	?	32/022/33342	*	1,1,1	*	absent	convergent
Gp 20	*P. khanka* sp. nov.	33/012/33342	1/000/100001^{m}	1,1,1	2+2	absent	parallel
Gp 21	?	32/022/33342	*	1,1,1	*	absent	convergent
Gp 22	*P.* cf. *taimyrica* (Martynova, 1976)	33/022/33342	1/000/110001^{m}	1,1,1	1+1	absent	parallel

Note: 1. Question mark means that the species cannot be given a name according to the present specimens that we have; asterisk means that this character status cannot be verified by the present specimens that we have; the character status of the deformities individuals are not included here. 2. Legends: A. Number of pso on subcoxae I of legs I–III; B, Number of ventral mesothoracic chaetae; C, Chaeta s' on abdominal terga I–III and V; D, Straight lines passing through base of prespinal on abdominal tergum VI.

Table S7. Comparison and variation of the two new species having dorsal pso formula as 33/012/33342 and ventral psx as 1/000/100001^{m}.

	Protaphorura jilinensis **sp. nov. (20 specimens)**	***Protaphorura khanka*** **sp. nov. (20 specimens)**
Number of chaetae on Th. sternum II	1+1 (90%), 1+2 (10%)	2+2 (80%), 1+2 (10%), 1+1 (10%)
Number of chaetae on subcoxa 1 of leg II	7	7 (70%), 6 (30%)
Number of chaetae on subcoxa 1 of leg III	5 (50%), 6 (45%), 7 (5%)	5
Number of basal chaetae on tibiotarsi of leg I	3 (95%), 4 (5%)	3 (15%), 4 (85%)
Number of basal chaetae on tibiotarsi of leg II	5	5 (95%), 4 (5%)
Number of basal chaetae on tibiotarsi of leg III	4	4 (95%), 3 (5%)
Number of axial chaeta m0 on Abd. tergum IV	1 (60%), 2 (40%)	1 (85%), 2 (15%)
Axial chaeta m0 on Abd. tergum V	present (40%), absent (60%)	present (60%), absent (40%)
